# Droplet digital polymerase chain reaction-based quantification of circulating microRNAs using small RNA concentration normalization

**DOI:** 10.1038/s41598-020-66072-z

**Published:** 2020-06-02

**Authors:** Shalini Das Gupta, Xavier Ekolle Ndode-Ekane, Noora Puhakka, Asla Pitkänen

**Affiliations:** 0000 0001 0726 2490 grid.9668.1 A. I. Virtanen Institute for Molecular Sciences, University of Eastern Finland, PO Box 1627, FI-70211 Kuopio, Finland

**Keywords:** Molecular biology, miRNAs

## Abstract

Quantification of plasma microRNAs (miRNAs) as non-invasive disease biomarkers is subject to multiple technical variabilities. This study aimed to develop an optimized protocol for miRNA quantification from rodent plasma. We hypothesized that a fixed small RNA concentration input for reverse transcription (RT) reaction will provide better miRNA quantification than a fixed RNA volume input. For this, tail-vein plasma was collected from 30 naïve, adult male Sprague-Dawley rats. Plasma hemolysis was measured with NanoDrop-1000 and Denovix DS-11 spectrophotometers. Plasma was then pooled, and RNA was extracted from 50-μl, 100-μl or 200-μl pool aliquots. Small RNA concentration was measured with Qubit miRNA assay. A fixed RNA volume (un-normalized) or a fixed small RNA concentration was used for RT (concentration-normalized). The method was setup with miR-23a-3p and validated with miR-103a-3p and miR-451a. Hemolysis measurements from Denovix and NanoDrop strongly correlated. Qubit revealed increased small RNA concentrations with increasing starting plasma volumes. With concentration-normalization, miRNA levels from 100-µl and 200-µl plasma volume groups mostly normalized to the level of the 50-µl in ddPCR. Our results indicate that miRNA quantification with ddPCR should be performed with small RNA concentration-normalization to minimize variations in eluted RNA concentrations occuring during RNA extraction.

## Introduction

MicroRNAs (miRNAs) are small non-coding RNAs (~22 nucleotides long) that regulate the expression of protein-coding genes through translational repression^1^. Dysregulation of miRNA levels is observed in many disease conditions, both in the affected tissue and the circulation^2–5^. Circulating serum/plasma miRNAs are extremely stable under appropriate sampling and storage conditions^6–10^, and are a potential source of non-invasive diagnostic, prognostic, and predictive biomarkers of disease states^11,12^.

Despite the stability of circulating plasma miRNAs, there are several challenges associated with their reliable and reproducible quantification as biomarkers. First, hemolysis in plasma impairs the detection of circulating cell-free miRNAs^6,9,13^. Second, the total amount of small RNA in the plasma is usually low, making it challenging to obtain accurate concentration measurements using common methods like the NanoDrop or Agilent Bioanalyzer^14^. Third, there are no endogenous normalizer miRNAs that can be used as an internal standard^15^. Some attempts have been made to overcome these challenges. For example, multiple methods are proposed for detecting plasma hemolysis^16–18^. The need to carefully report the protocols used for blood collection, centrifugation, storage, and handling of plasma has been emphasized, because these factors profoundly affect circulating miRNA levels, and the harmonization of these techniques is important for standardizing plasma biomarker research across laboratories^18–22^. The addition of carrier RNAs and synthetic spike-ins during RNA extraction and reverse transcription-quantitative polymerase chain reaction (RT-qPCR) is also useful^10,23,24^. Further, fluorometric assays such as Qubit have been developed to quantify small RNA concentrations from low-abundance samples with higher specificity and sensitivity than the NanoDrop or Bioanalyzer^14^. To circumvent the problem of normalization, absolute quantification methods such as droplet digital PCR (ddPCR) have been developed^25^.

One factor that remains to be addressed, however, is the use of a fixed RNA sample volume for cDNA synthesis due to the poor estimation of the eluted RNA concentration^22,26,27^. Eluted RNA concentrations from the same starting plasma volumes might differ due to sample-to-sample variations in RNA concentrations that occur during extraction. Thus, with fixed plasma/serum volumes, normalization of ddPCR data remains a requirement.

In this work, we examined the sample volume normalization issue. As plasma is preferred over serum for miRNA biomarker analysis^18,28^, we focused on rodent plasma samples as a pre-clinical scenario. We selected miR-23a-3p to setup a method for a-priori small RNA concentration normalization for cDNA synthesis, instead of using fixed RNA sample volumes. We selected miR-23a-3p because of its relatively stable expression abundance in the plasma of healthy humans^16^ and naïve rats^18,19^. To validate the method, we analysed plasma miR-103a-3p and miR-451a levels, since these miRNAs also have an abundant expression in human and rat plasma^16,29^. Adding to our previous knowledge^18,19^, we first optimized our hemolysis measurement protocol to select the most suitable rat tail-vein plasma aliquots for miRNA biomarker analysis. Next, we measured the eluted small RNA concentrations using the Qubit miRNA assay and evaluated the assay performance. In addition to assessing the fixed RNA sample volume input, we also assessed a fixed small RNA concentration input for cDNA synthesis, based on the Qubit measurements. Finally, we performed absolute quantification of miR-23a-3p, miR-103a-3p and miR-451a from both the volume-normalized and small RNA concentration-normalized samples using ddPCR. Based on the data from setup and validation phases, we propose that plasma miRNA biomarker analysis can be performed from a fixed volume of plasma, followed by small RNA concentration-normalization at the reverse transcription (RT) step and absolute quantification of the target miRNA copy number with ddPCR.

## Materials and methods

### Animals

**Figure 1:**
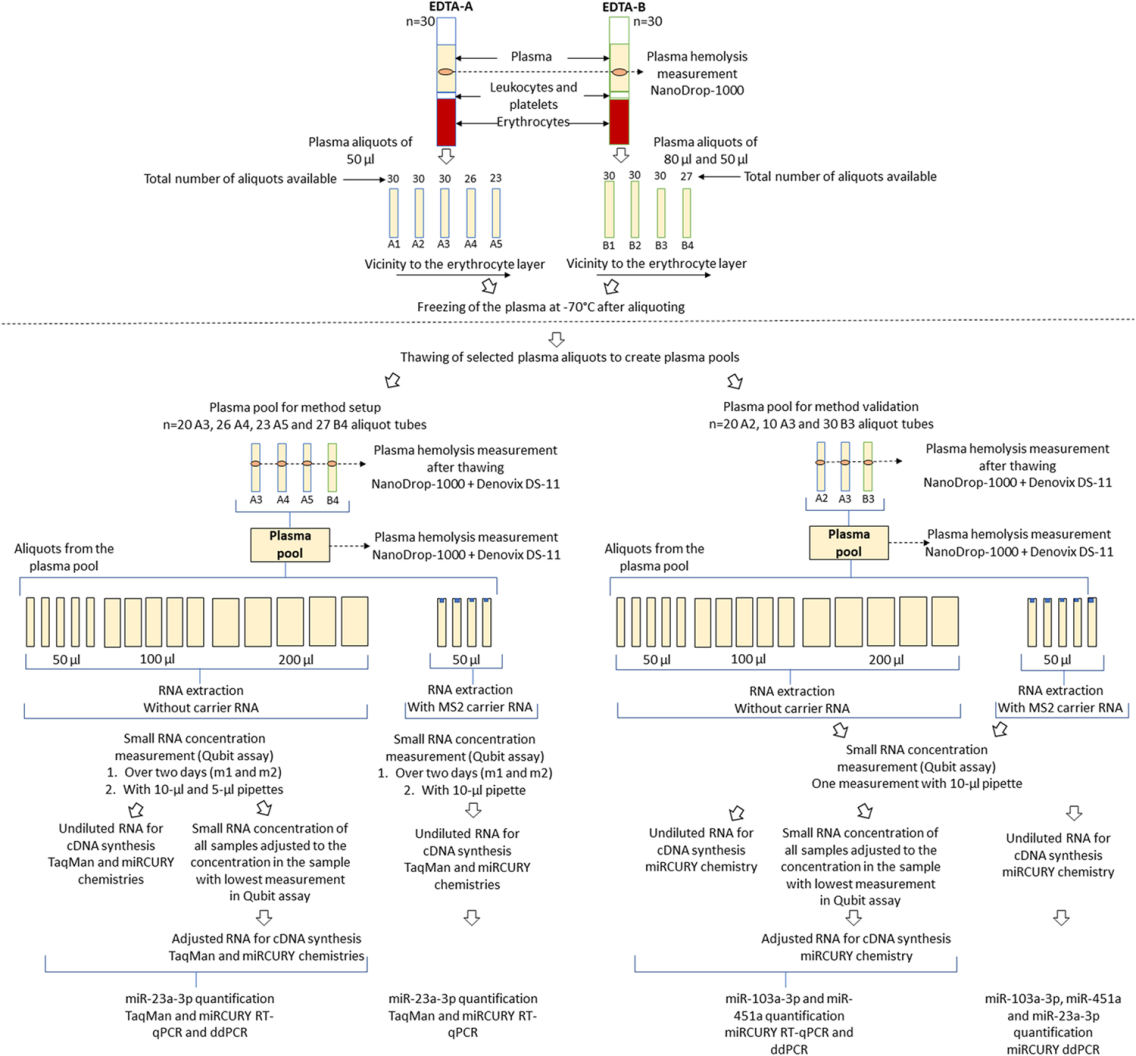
Flow-chart of the study design. A flow-chart describing the preparation of the plasma pools and the steps involved in small RNA concentration normalization method setup and validation. Abbreviations: cDNA, Complimentary DNA; ddPCR, Droplet digital polymerase chain reaction; EDTA, Ethylenediaminetetraacetic acid; m1, Qubit small RNA concentration measurement on day 1; m2, Qubit small RNA concentration measurement on day 2; RT-qPCR, Reverse transcriptase-quantitative polymerase chain reaction.

The animal numbers and study design are summarized in Fig. 1. Adult naïve male Sprague-Dawley rats (n = 30, 12 weeks of age at the time of blood sampling, mean weight 317 g, Envigo B.V., Netherlands) were used in this study. The rats were housed in a controlled environment (temperature 22 ± 1 °C; humidity 50–60%; lights on from 07:00–19:00 h). Water and pellet food were provided ad libitum. All animal procedures were approved by the Animal Ethics Committee of the Provincial Government of Southern Finland and carried out in accordance with the guidelines of the European Community Council Directives 2010/63/EU.

### Plasma sampling from tail-vein, hemolysis measurement, and creation of the plasma pools

#### Plasma sampling

Tail-vein plasma was sampled from the rats (n = 30) according to the 3Rs (www.nc3rs.org.uk/rat-tail-vein-non-surgical) and following our previously optimized protocol^18,19^. Briefly, 1 ml of whole blood was drawn from the lateral tail vein under isoflurane anesthesia (induction: 5%, maintenance: 1–2%) into two Microtainer K_2_ EDTA-tubes (di-potassium ethylenediaminetetraacetic acid, Microtainer, BD Biosciences, Franklin Lakes, NJ, 500 μl/tube), using a 25 G butterfly needle (Surflo Winged infusion set, Terumo Europe N.V., Leuven, Belgium). The blood samples were thoroughly mixed with EDTA and immediately placed on ice. For each animal, the two K_2_ EDTA-tubes were labelled as EDTA-A and EDTA-B. Tube A was always filled first followed by tube B. Whole blood samples were centrifuged at 1300 g (Centrifuge 5417 R, Eppendorf Biotools CA) for 10 min (+4 °C) to obtain plasma. The hemolysis coefficient of the plasma was measured at 414 nm (UV-Vis module), using a NanoDrop-1000 spectrophotometer. For this measurement, 1.5 μl of plasma was pipetted approximately from the middle of the plasma layer in the EDTA tube.

The plasma was then aliquoted into Protein LoBind tubes (Eppendorf LoBind, Eppendorf AG, Hamburg, Germany). Plasma from the EDTA-A tube was aliquoted in five 50-μl aliquots (labelled A1-A5), whereas the plasma in the EDTA-B tube was aliquoted into two 80-μl aliquots (labelled B1-B2) and two 50-μl aliquots (labelled B3-B4). Manual notes were prepared by an experienced researcher pipetting the aliquots, regarding (a) observable red blood cell contamination, (b) if the pipetted volume in the Eppendorf tube was less than intended, and (c) if the last aliquoted tubes were not complete due to a smaller plasma volume obtained than anticipated. The aliquots were then frozen in dry ice and stored at −70 °C until processed.

#### Generation of the plasma pools

For method setup, a plasma pool was prepared by combining plasma from all available A4, A5 and B4 aliquots (26 A4, 23 A5, and 27 B4 tubes), and 20 A3 aliquots from the first 20 rats of the cohort (96 aliquot tubes in total, plasma volume ~3 ml). For the validation phase, a second plasma pool was prepared using all available B3 aliquots (30 B3 tubes), the remaining 10 A3 aliquots and 20 A2 aliquots from the first 20 rats of the cohort (60 aliquot tubes in total, plasma volume ~3 ml). The remaining A1-A2 and B1-B2 aliquot tubes were preserved for future studies. For creating the plasma pools, frozen aliquots were thawed on ice and the hemolysis of each individual aliquot was measured with the NanoDrop-1000 and Denovix DS-11 spectrophotometers. Thawed plasma from all tubes were pooled and hemolysis in the total plasma pools were again measured with the NanoDrop-1000 and Denovix DS-11. Finally, various volumes of aliquots were taken from the plasma pools for RNA extraction.

### Method setup

#### RNA extraction from plasma

##### Total RNA extraction without carrier RNA

Total RNA was extracted from five 50-μl aliquots, five 100-μl aliquots, and five 200-μl aliquots of the first plasma pool using the miRNeasy serum/plasma protocol (Qiagen, Germany, https://www.qiagen.com/fi/resources/resourcedetail?id=1076a54d-7967-4bc0-a34e-e3f574641d92&lang=en). The protocol was slightly modified: the addition of the spike-in control was omitted, and final elution of RNA was performed in 30 μl nuclease-free water, instead of 14 μl. The same RNA isolation protocol was also applied to a 50-μl water sample as a no-template control. RNA elutions were used for miR-23a-3p quantification with the TaqMan and miRCURY chemistries.

Total RNA extraction with MS2 carrier RNA. To check if the addition of a carrier RNA improves downstream miRNA quantification, we isolated total RNA from another four 50-μl aliquots of the same plasma pool, using the miRNeasy serum/plasma protocol (Qiagen, Germany). After pipetting 250 μl Qiazol lysis reagent into each plasma sample, we added 1 μg of MS2 bacteriophage carrier RNA (#10165948001, Roche Diagnostics GmbH, Mannheim, Germany) into each tube. We performed an additional wash step with RPE buffer (provided in the miRNeasy serum/plasma kit)^24^. The same RNA isolation protocol with the addition of the MS2 carrier RNA was also applied to a 50-μl water sample to obtain a no-template control, as described previously^24^. Also, in the carrier-added samples, miR-23a-3p quantification was performed with the TaqMan and miRCURY chemistries.

Measurement of small RNA concentration with the Qubit microRNA assay kit. For the 15 plasma RNA samples extracted without carrier, small RNA concentrations in the RNA eluates were measured with the Qubit miRNA assay kit (Catalog no. Q32880, ThermoFisher Scientific). We investigated if one freeze-thaw cycle of the eluted RNA affected the small RNA concentration measurements in Qubit. We also investigated if a difference in the choice of pipettes used to add the RNA eluate to the Qubit assay affected the assay variability, as noted in an application note from Thermofisher Scientific for Qubit assays^30^. For this, measurements were performed (a) immediately after RNA extraction (m1) and 1 day later (m2) after a single freeze-thaw cycle of the RNA. Further, each of these measurements were performed using (a) either a 10-μl pipette (#725020, Sartorius mLINE mechanical pipette, 1-ch, 0.5–10 µl, Sartorius, Finland) or (b) a 5-μl pipette (#4642100, Thermo Scientific Finnpipette, 0.5–5 µl, Thermo Scientific, Finland) to pipette the RNA eluate to the Qubit assay. Because the RNA elution volume was limited to 30 μl, we used the traditional forward pipetting method instead of the reverse pipetting method. The RNA volume added to the Qubit reaction was limited to 1 μl to maintain the required amount of eluted RNA for downstream RT-qPCR and ddPCR applications.

For the plasma RNA samples with the carrier RNA, Qubit measurements were obtained as for the samples without the carrier, but only the 10-μl pipette was used as our data indicated no difference in performance between the two pipettes (see Results).

#### Reverse transcription of RNA to cDNA

Reverse transcription from samples with un-normalized small RNA concentration. A fixed volume of RNA eluate from the different starting plasma volume groups (50-μl, 100-μl, and 200-μl) was used as input for the RT reaction.

TaqMan chemistry. Total RNA (with and without carrier RNA) was transcribed to complimentary DNA (cDNA) with the TaqMan miRNA Reverse Transcriptase Kit (Catalog no. 4366596, ThermoFisher Scientific, https://assets.thermofisher.com/TFS-Assets/LSG/manuals/4364031_TaqSmallRNA_UG.pdf) according to the manufacturer’s instructions. Samples were stored at −20 °C until further processed.

miRCURY chemistry. Total RNA (with and without carrier RNA) was transcribed to cDNA with the miRCURY LNA RT Kit (Catalog no. 339340, Qiagen, https://www.qiagen.com/fi/resources/resourcedetail?id=7ab5f614-f5d6-4bdc-b22b-246ec3601588&lang=en) according to the manufacturer’s instructions. Samples were stored at −20 °C until further processed.

### Reverse transcription from samples with normalized small RNA concentration

To normalize the small RNA concentration, a fixed small RNA concentration was used as input for the RT reaction from the samples of the different starting plasma volume groups (50-μl, 100-μl, and 200-μl). For this, small RNA concentrations of the 15 plasma samples isolated without carrier RNA were adjusted to the concentration in the sample with the lowest concentration of small RNAs measured with Qubit. Thus, the RNA sample with the lowest concentration was used undiluted in the cDNA reaction. The other samples were then diluted with nuclease-free water to adjust their concentration of small RNA applied to the cDNA reaction to be comparable to that of the undiluted sample. cDNA synthesis was performed using the TaqMan and miRCURY chemistries as described above. Small RNA concentration normalization was not performed for the carrier added samples, since Qubit did not accurately estimate the concentrations in the presence of carrier (see Results).

### Analysis of circulating miR-23a-3p levels with RT-qPCR and ddPCR

The PCR reaction setups and thermal cycling conditions used for plasma miR-23a-3p quantification are summarized in Supplementary Table S10.

### RT-qPCR of plasma miR-23a-3p

#### TaqMan chemistry

The plasma miR-23a-3p level was determined from both un-normalized and normalized cDNA samples (without carrier RNA) using the TaqMan Small RNA Assays protocol (https://assets.thermofisher.com/TFS-Assets/LSG/manuals/4364031_TaqSmallRNA_UG.pdf). For samples with carrier RNA, the analysis was performed only from un-normalized cDNA.

#### miRCURY chemistry

Plasma miR-23a-3p levels from the un-normalized and normalized cDNA samples (without carrier) were also analyzed with miRCURY chemistry. For samples with carrier RNA, the analysis was performed only from un-normalized cDNA. RT-qPCR reactions were performed according to the protocol (https://www.qiagen.com/fi/resources/resourcedetail?id=7ab5f614-f5d6-4bdc-b22b-246ec3601588&lang=en). cDNA samples were diluted 30-fold with nuclease-free water for the RT-qPCR reaction.

#### RT-qPCR of the spike-in UniSp6 with miRCURY chemistry

To monitor the efficiency of the RT step, RT-qPCR was also performed using the un-normalized and normalized cDNA samples (without carrier RNA) for the synthetic spike-in UniSp6 added during the miRCURY RT reaction. RT-qPCR was performed in the same way as described above for miR-23a-3p, except that the UniSp6 primer assay (included in the miRCURY LNA miRNA PCR Starter kit, Catalog no. 339320, Qiagen) was used.

### ddPCR of plasma miR-23a-3p

#### TaqMan chemistry

For ddPCR, 1.33 μl of un-normalized and normalized cDNA templates from the TaqMan RT reaction (without carrier RNA, same samples as used for RT-qPCR) were added to a 20-μl reaction mixture containing 10 μl Bio-Rad 2x ddPCR Supermix for probes (#186-3010, Bio-Rad), 1 μl 20x miR-23a-3p PCR primer, and 7.67 μl nuclease-free water. DdPCR reaction was performed as described previously^31^. For each sample, the reaction was performed in duplicate, and the mean of miR-23a-3p copies/20-μl PCR reaction from the two replicates was calculated and used for representation in the figures (with automatic amplitude threshold). No ddPCR was performed for the samples containing carrier RNA.

#### miRCURY chemistry

For miRCURY ddPCR, we tested the un-normalized cDNA under several conditions (miRCURY ddPCR setups 1–6, Supplementary Table S10). We compared the results by evaluating the correlation between the mean miR-23a-3p copies and (i) the Qubit concentrations, (ii) the mean miR-23a-3p Cq cycles (Supplementary Table S12). For these conditions, the 20-μl reaction mixtures were prepared with 10 μl Bio-Rad 2x ddPCR EvaGreen Supermix (#186-4034, Bio-Rad), 1–2 μl of the miR-23a-3p PCR primer, 1–3 μl nuclease-free water, and 6–8 μl of diluted un-normalized cDNA templates from the miRCURY RT reaction (without carrier, same samples as used for the RT-qPCR). Droplets were generated the same way as described above, with 70 μl of droplet generation oil for EvaGreen (#186-4005, Bio-Rad). Based on the results, two of the tested conditions were selected for application to the normalized cDNA templates: (i) miRCURY ddPCR setup 3 and (ii) miRCURY ddPCR setup 6^32^. No ddPCR was performed for the samples containing carrier RNA.

### Method validation

To validate the method, total RNA was extracted from five 50-μl aliquots, five 100-μl aliquots, and five 200-μl aliquots of the second plasma pool using the miRNeasy serum/plasma protocol, as described previously. In addition, total RNA was also isolated from another five 50-μl aliquots of the same plasma pool with the MS2 carrier addition. The additional wash step with RPE was performed for the carrier-added samples, as done previously. Eluted small RNA concentrations were measured with the Qubit miRNA assay kit using only the 10-μl pipette. Additionally, measurements were performed only once (after one freeze-thaw cycle of the RNA), since the setup phase indicated no difference in measured small RNA concentrations over different days or with using different pipettes (see Results). Small RNA concentration normalization was performed as described above. Plasma levels of miR-103a-3p (Catalog no: YP00204063, Qiagen) and miR-451a (Catalog no: YP02119305, Qiagen) were analyzed from the un-normalized and small RNA concentration-normalized samples using miRCURY RT-qPCR and ddPCR. For this, RT reaction products were diluted 30-fold for miR-103a-3p, and 60-fold for miR-451a with nuclease-free water. The miRCURY chemistry was chosen since (i) it requires smaller RNA volume for PCR analysis due to the universal RT reaction in comparison to the miRNA-specific RT reaction in TaqMan, (ii) the setup phase indicated comparable performance of the two chemistries (see Results). Further, since miRCURY ddPCR setup 6 performed most optimally in the setup phase (see Results), we chose only that setup for miR-103a-3p and miR-451a analysis. In the validation phase, miR-103a-3p, miR-451a levels and miR-23a-3p were also analyzed with miRCURY ddPCR from the un-normalized samples with carrier RNA.

### Statistical analysis

Statistical analyses were performed using IBM SPSS Statistics 25.0 (IBM Corp., Armonk, NY). Graphs were prepared with GraphPad Prism (version 8.0.1, GraphPad Software, San Diego, CA). The non-parametric Kruskal-Wallis test was used for comparison of three or more independent groups, and the Mann-Whitney *U* test was applied for *post hoc* analysis^33^. For three or more related samples, the Friedman test was used. The Wilcoxon signed rank test was used for pairwise comparisons. Correlations were analyzed based on Spearman’s rho (ρ). A P-value less than 0.05 was considered statistically significant. All the statistical tests performed in this study are summarized in Supplementary Table S16.

## Results

### Hemolysis coefficients of the EDTA tubes, individual plasma aliquots, and pooled plasma– comparison of the Nanodrop, Denovix and visual analysis

#### EDTA tubes

An absorbance value >0.25 at 414 nm in the NanoDrop measurement was used as a cut-off for defining whether or not the sample was hemolysed^13,17,18^. NanoDrop-1000 analysis of the EDTA-A and EDTA-B tubes immediately after centrifugation indicated that 1 of the 30 EDTA-A tubes and none of the 30 EDTA-B tubes contained a hemolysed sample (Supplementary Table S1).

#### Plasma aliquots

We then compared the NanoDrop-1000 and Denovix DS-11 instruments for measuring hemolysis in the individual A2, A3, A4, A5, B3 and B4 plasma aliquots that were pipetted from the centrifuged EDTA tubes, with later aliquots being closer to the erythrocyte layer than earlier aliquots. Based on a cut-off absorbance value of >0.25 in the NanoDrop assay, the cut-off threshold for the Denovix (Denovix limit_value_) was calculated using the following formula:

$$Denovix\,limi{t}_{value}(A)=NanoDrop\,limi{t}_{value}(A)\ast \left(\frac{\varSigma \left(\frac{Denovix(A)}{NanoDrop(A)}\right)}{n(samples)}\right)$$

where NanoDrop limit_value_(A) = 0.25, Denovix(A) = absorbance value of each plasma aliquot measured with Denovix DS-11, NanoDrop(A) = absorbance value of each plasma aliquot measured with NanoDrop-1000, n(samples) = total number of plasma aliquots measured. With this equation, the cut-off threshold for detecting hemolysis in the plasma samples with the Denovix DS-11 was defined as >2.5.

**Figure 2:**
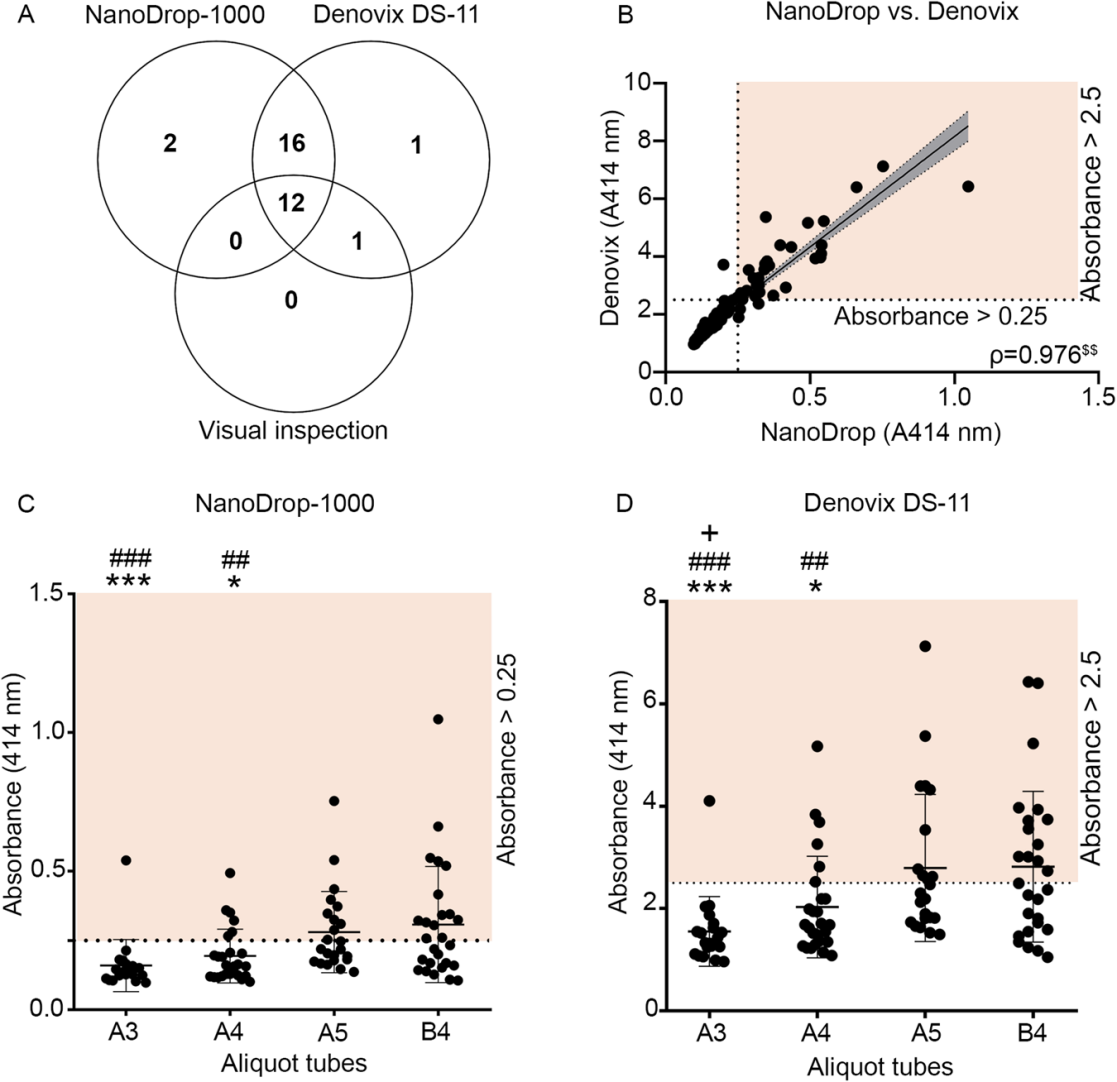
Plasma hemolysis measurements with NanoDrop-1000, Denovix DS-11 and visual analysis in the setup phase. (**A**) With all the three methods, 12/96 aliquots were detected to be hemolysed. **(B)** NanoDrop and Denovix measurements were highly correlated (Spearman rho (ρ) = 0.976, p < 0.01). **(C)** The A4 aliquots had lower mean hemolysis coefficients than B4 (p < 0.05) and A5 (p < 0.01). Similarly, A3 aliquots had lower mean hemolysis coefficients than B4 (p < 0.001) and A5 (p < 0.001). **(D)** The Denovix and Nanodrop showed very similar patterns. The Denovix also showed lower absorbance in the A3 tubes compared with A4 (p < 0.05). The orange shaded area in panels **(C)** and **(D)** indicate the samples with hemolysis absorbance values >0.25 or >2.5 in the NanoDrop and Denovix, respectively. Statistical significances: *p < 0.05 and ***p < 0.001 compared to B4; ^##^p < 0.01 and ^###^p < 0.001 compared to A5; + p < 0.05 compared to A4; ^$$^p < 0.01 in Spearman’s correlation. Abbreviations: A3, the 3^rd^ 50-μl plasma aliquot from the EDTA-A tube; A4, the 4^th^ 50-μl plasma aliquot from the EDTA-A tube; A5, the 5^th^ 50-μl plasma aliquot from the EDTA-A tube; B4, the 4^th^ 50-μl plasma aliquot from the EDTA-B tube.

For the plasma aliquots used in method setup, hemolysis was visually detected in 13.5% (13/96) of the tubes. When the aliquots were thawed and a second hemolysis measurement was performed using NanoDrop and Denovix, hemolysis was detected in 29% (28/96) of all samples by at least one method (A414nm > 0.25 or >2.5 respectively) (Fig. 2A ^34^; Supplementary Tables S2-S5). With both instruments and visual detection, hemolysis was detected in 12.5% (12/96) of the plasma aliquots (Fig. 2A). The hemolysis measurements by the two devices were very strongly and positively correlated (ρ = 0.976, p < 0.01) (Fig. 2B).

We then assessed the effect of the aliquoting order on the presence of hemolysis. As expected, the last aliquots from both the EDTA-A and EDTA-B tubes, i.e., the A5 and B4 tubes, exhibited more hemolysis than the A3 and A4 tubes. Among the A3, A4, A5 and B4 tubes, hemolysis was detected in 5% (1/20), 23% (6/26), 39% (9/23) and 44% (12/27) of the tubes respectively, with both instruments (Supplementary Table S2-S5).

Analysis of the mean hemolysis coefficient indicated no difference between the last aliquots (A5 and B4; p > 0.05) detected by either instrument. With both instruments, however, the hemolysis coefficients were lower for the A4 tubes than for the A5 (p < 0.01) or B4 (p < 0.05) tubes. The hemolysis coefficients for the A3 tubes were also lower than those for the A5 (p < 0.001) or B4 tubes (p < 0.001). With the NanoDrop, the hemolysis coefficients did not differ between the A3 and A4 tubes (p > 0.05). The Denovix, however, revealed less hemolysis in the A3 tube than in the A4 tube (p < 0.05; Fig. 2C,D).

In the method validation phase, a new plasma pool was created using the A2, A3 and B3 aliquots. Since these were the first three aliquots pipetted from the corresponding EDTA tubes and were farther away from the erythrocyte layer, hemolysis was low in these aliquots. Hemolysis was visually detected in 6.7% (4/60) of the tubes. When the aliquots were thawed and hemolysis was again measured with NanoDrop and Denovix, NanoDrop detected hemolysis in 8.3% (5/60) of the aliquots (A414nm > 0.25) and Denovix detected hemolysis in 3.3% (2/60) of the aliquots (A414nm > 2.5) (Supplementary Fig. 4A^34^; Supplementary Tables S6-S8). With both instruments and visual detection, hemolysis was detected in 3.3% (2/60) of the plasma aliquots. As expected, hemolysis measurements by the two devices were very strongly and positively correlated (ρ = 0.936, p < 0.01) (Supplementary Fig. 4D). Analysis of the mean hemolysis coefficients indicated no difference between the A2, A3 and B3 aliquots (p > 0.05; Supplementary Fig. 4B,C).

Hemolysis measured from the EDTA tubes (before freezing) were also compared with that of the individual aliquot tubes (after melting). Since hemolysis was measured before freezing with NanoDrop-1000, comparisons were performed only based on NanoDrop-1000 measurements. There was correlation between the EDTA-B tube and its respective aliquots (B3 and B4) but not for the EDTA-A tube and its aliquots (Supplementary Table S9). However, for both EDTA tubes, hemolysis values in aliquots increased with increased vicinity to erythrocyte layer (Supplementary Fig. 1).

### Pooled plasma

Hemolysis values for the plasma pool used for method setup were 0.172 and 1.635 with NanoDrop and Denovix respectively. The plasma pool used for validation had hemolysis values of 0.144 and 1.34 with NanoDrop and Denovix respectively. As the values were below the cut-off thresholds for both methods, the plasma pools were considered suitable for downstream analysis.

### Measurement of small RNA concentrations with Qubit

#### RNA extractions without carrier RNA

**Figure 3:**
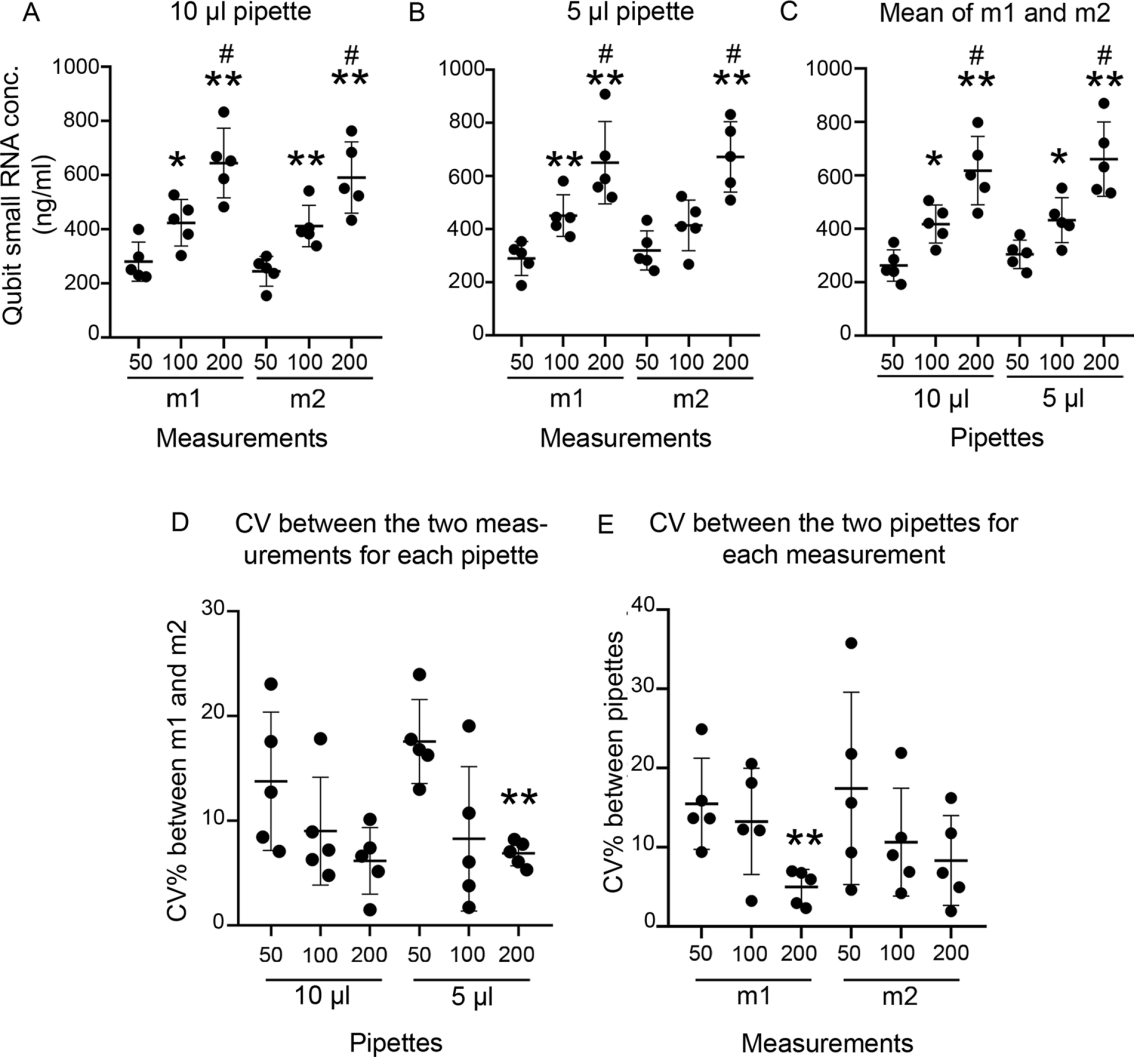
Qubit miRNA assay revealed increase in small RNA concentrations with increase in starting plasma volumes in the setup phase. Small RNA concentrations were measured from the RNA eluates with the Qubit assay. For this, 1 μl of RNA eluate from each sample was pipetted to the assay using either a 10-μl pipette or a 5-μl pipette. Two measurements were performed from each sample involving one freeze thaw cycle of the RNA (m1 and m2). **(A)** With the 10-μl pipette, both 100-μl and 200-μl groups had higher small RNA concentration than the 50-μl. **(B)** A similar pattern was observed with the 5-μl pipette. However, concentrations in the 100-μl group were higher than those in the 50-μl group only in m1. **(C)** For mean small RNA concentrations from m1 and m2, both pipettes revealed increased small RNA concentrations with increase in starting plasma volume. **(D)** Use of the 5-μl pipette revealed a lower CV% between m1 and m2 for the 200-μl group than the 50-μl group. **(E)** CV% between m1 from 10-μl pipette and 5-μl pipette was also lower in the 200-μl group in comparison to the 50-μl group. Statistical significances: *p < 0.05 and **p < 0.01 compared with the 50 μl plasma volume group within the same pipette type or measurement, ^#^p < 0.05 compared with the 100-μl plasma volume group within the same pipette type or measurement. Abbreviations: conc., Concentration; CV, Coefficient of variation; m1, Qubit small RNA concentration measurement in day 1, m2, Qubit small RNA concentration measurement on day 2.

During method setup, eluted small RNA concentrations were measured with Qubit, using either a 10-μl pipette or a 5-μl pipette to dispense 1 μl of RNA eluate for the Qubit assay. With the 10-μl pipette, we observed that an increase in the small RNA concentration corresponded to an increase in the starting plasma volume. For both the first measurement (m1, before the freeze-thaw cycle) and the second measurement (m2, after one freeze-thaw cycle), the amount of small RNA eluted from the 100-μl plasma aliquot was greater than that obtained from the 50-μl aliquot (m1, p < 0.05; m2, p < 0.01). Similarly, the amount of small RNA eluted from 200-μl aliquots was greater than that from the 50-μl (p < 0.01 in both m1 and m2) and 100-μl (p < 0.05 in both m1 and m2) aliquots. For each starting plasma volume, the amount of small RNA measured did not differ between m1 and m2 (Fig. 3A). Very similar results were obtained with the 5-μl pipette (Fig. 3B). When the mean of m1 and m2 was calculated, an increase in the concentration was observed with an increase in the starting plasma volume for both pipettes (Fig. 3C). The concentration of small RNA detected in the nuclease-free water sample was below the limits of detection.

The percentage of coefficient of variation (CV%) between the two measurements from each pipette was calculated for every plasma volume group (CV% between m1 and m2 from 10-μl pipette; CV% between m1 and m2 from 5-μl pipette; Fig. 3D). CV% between each measurement from the two pipettes was also calculated for every plasma volume group (CV% between m1 from 10-μl and 5-μl pipettes; CV% between m2 from 10-μl and 5-μl pipettes; Fig. 3E). CV% was calculated using the following formula: [((STDEV(m1,m2)/AVERAGE(m1,m2)))*100]. For the 5-μl pipette, the CV% between m1 and m2 was smaller in the 200-μl group compared with the 50-μl group (p < 0.01; Fig. 3D). CV% between m1 from the two pipettes was smaller for the 200-μl group compared with the 50-μl group (p < 0.01; Fig. 3E).

When the correlation of the small RNA concentrations measured by the 10-μl and the 5-μl pipettes was analyzed for m1, m2, and the mean of the two measurements, only the small RNA concentrations from the 200-μl plasma group correlated for all three (p < 0.01 in m1; p < 0.05 for m2 and the mean of m1 and m2; Supplementary Fig. S2). When the correlation of the small RNA concentrations from m1 and m2 was calculated for each pipette, m1 and m2 correlated for the 200-μl plasma group with both the 10-μl pipette (p < 0.01) and the 5-μl pipette (p < 0.05). M1 and m2 also correlated for the 100-μl group with the 5-μl pipette (p < 0.05; Supplementary Fig. S3).

Because both pipettes showed very similar trends and CV% in Qubit small RNA concentration measurements and the 10-μl pipettes are more commonly available in laboratories, we considered the measurements from this pipette for downstream miRNA quantification. The mean small RNA concentration of m1 and m2 from the 10-μl pipette was considered as the final concentration in each sample. With this, we identified that the 100-μl and 200-μl plasma volume groups had only 1.6-fold and 2.4-fold higher small RNA concentrations than the 50-μl group rather than the 2-fold and 4-fold increases, respectively, that we expected.

In the method validation phase, small RNA concentrations were measured only with the 10-μl pipette, and after one freeze thaw cycle of the RNA. As expected, an increase in the small RNA concentration corresponded to an increase in the starting plasma volume. The amount of small RNA eluted from the 100-μl plasma aliquots was greater than that obtained from the 50-μl aliquots (p < 0.01). Similarly, the amount of small RNA eluted from 200-μl aliquots was greater than that from the 50-μl and 100-μl aliquots (p < 0.01; Supplementary Fig. 4E). The 100-μl and 200-μl plasma volume groups had 1.6-fold and 3.0-fold higher small RNA concentrations than the 50-μl group in this phase.

#### RNA extractions with carrier

For the RNA samples extracted with MS2 carrier (both in setup and validation phase), Qubit measurement was performed only with the 10-μl pipette due to the comparable performance of the two pipettes. For these samples, Qubit detected a very high concentration. When the concentrations were compared with corresponding elutions from 50-μl plasma without carrier, a 13-fold increase in detection was obtained in the setup phase (mean concentration: 3340.8 ng/ml vs. 262.6 ng/ml). Surprisingly, for the control sample where RNA extraction was performed from 50-μl of nuclease-free water with MS2 carrier RNA added during the extraction process, Qubit also detected a small RNA concentration of 3154.4 ng/ml. This indicated that for carrier-added samples, Qubit detects the carrier RNA, and the real eluted small RNA concentration is masked. Similar results were obtained in the validation phase. A 33-fold increase in small RNA concentration was measured with Qubit from the carrier added samples, in comparison to the corresponding elutions from 50-μl plasma without carrier (mean concentration: 4968.7 ng/ml vs. 150.5 ng/ml). Thus, small RNA concentration normalization was not performed for these samples in any of the two phases.

### Comparison of miR-23a-3p, miR-103a-3p and miR-451a RT-qPCR from un-normalized and concentration-normalized RNA samples

In the setup phase, only miR-23a-3p was analysed. In the validation phase, miR-103-3p and miR-451a levels were analysed.

#### TaqMan chemistry for RNA extractions without carrier (setup phase)

**Figure 4:**
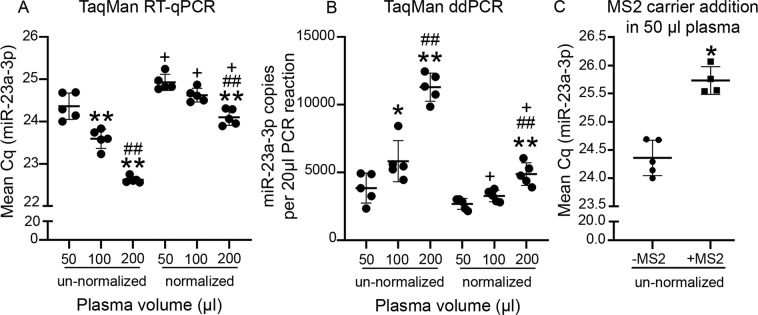
Concentration-normalization in setup phase revealed similar miR-23a-3p levels in the 50-μl and 100-μl groups with TaqMan chemistry. **(A)** With un-normalized samples, mean miR-23a-3p Cq decreased with corresponding increase in starting plasma volumes. For the normalized samples, mean miR-23a-3p Cq was similar between the 50-μl and 100-μl groups. The 200-μl group still had a lower mean Cq than either of the other groups. As a result of normalization, the mean miR-23a-3p Cq for each of the normalized plasma volume groups was higher than the corresponding un-normalized counterpart. **(B)** With ddPCR, the expression pattern was similar to that of RT-qPCR. The mean miR-23a-3p copy number per 20 μl of PCR reaction volume did not differ between the unnormalized and normalized 50 μl group. **(C)** Addition of the MS2 carrier RNA did not improve the miR-23a-3p yield. Statistical significances: *p < 0.05 and **p < 0.01 compared to 50 μl within normalized or un-normalized conditions; ^##^p < 0.01 compared to 100 μl within normalized or un-normalized conditions; +p < 0.05 normalized group compared to the same volume from the un-normalized group. Abbreviations: Cq, Cycle quantification; ddPCR, Droplet digital PCR; RT-qPCR, Reverse transcriptase quantitative polymerase chain reaction.

As a first step, RT-qPCR was performed from the un-normalized RNA. As expected, mean miR-23a-3p Cq decreased with a corresponding increase in the starting plasma volumes. Cq values were lower for the 100-μl plasma volume group than the 50-μl group (p < 0.01). Cq values were also lower for the 200-μl plasma volume group than the 50-μl or 100-μl group (p < 0.01 for both) (Fig. 4A). The mean Cq values correlated with Qubit small RNA concentrations for the 50-μl group (p < 0.05), but not for the 100-μl or 200-μl group (p > 0.05 for both) (Supplementary Table S11).

Next, we performed miR-23a-3p RT-qPCR from the small RNA concentration-normalized samples. We expected that with this normalization, all samples would exhibit similar Cq values for miR-23a-3p irrespective of the starting plasma volumes, because the small RNA input for the cDNA reactions was the same for all samples. The normalized 100-μl plasma volume group had a similar Cq as the normalized 50-μl group (p > 0.05). The normalized 200-μl group still had a lower Cq than either the 50-μl or 100-μl group (p < 0.01 for both). As a result of the small RNA concentration-normalization, standard deviation (SD) and CV% within each group reduced for the 50-µl and 100-μl groups, but not for the 200-μl (Supplementary Table S15).

#### miRCURY chemistry for RNA extractions without carrier (setup phase)

**Figure 5:**
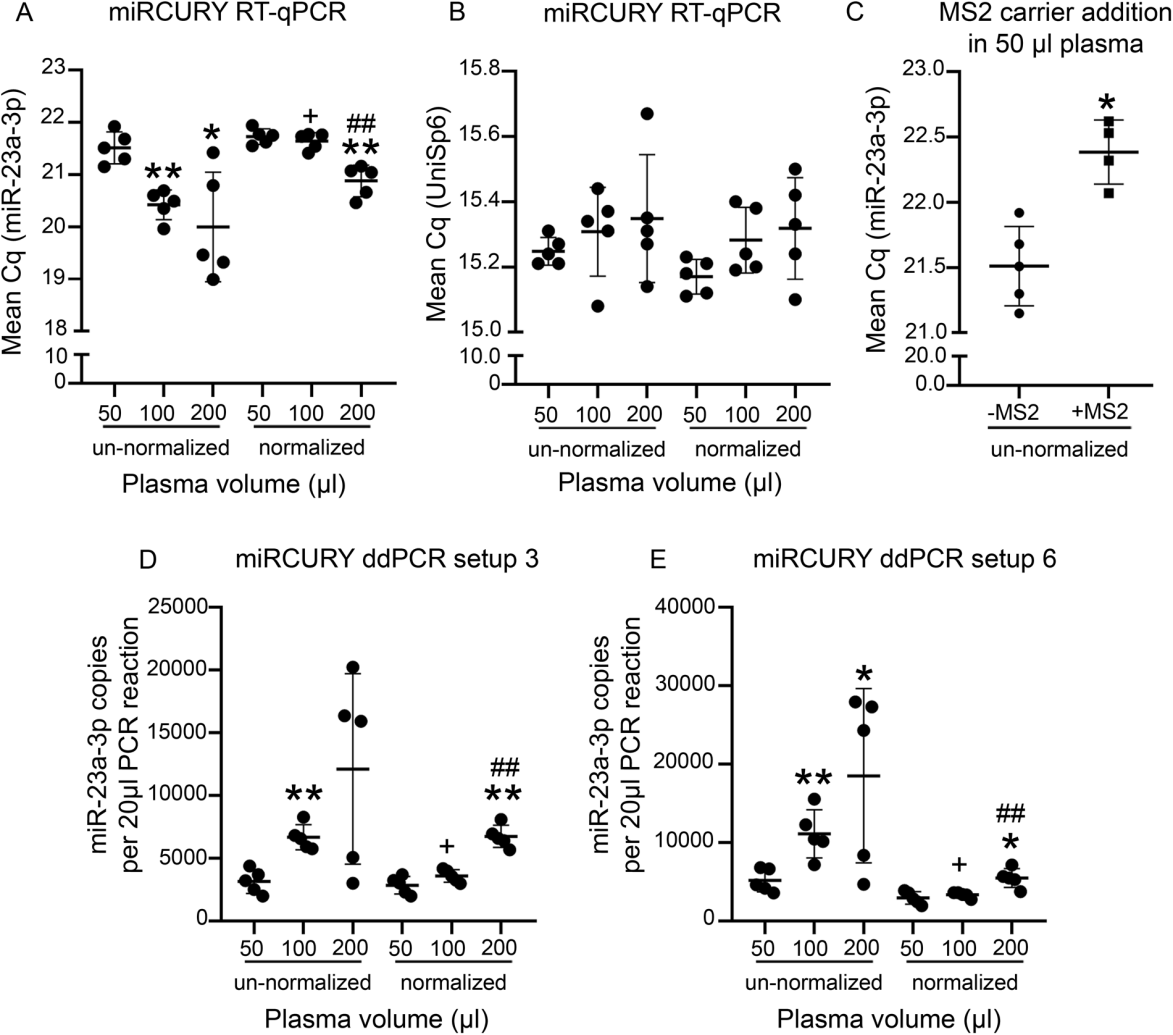
Concentration-normalization in setup phase revealed similar miR-23a-3p levels in the 50-μl and 100-μl groups with miRCURY chemistry. **(A)** With un-normalized samples, mean miR-23a-3p Cq was lower in the 100-μl group than in the 50-μl group. The 200-μl group had a lower mean Cq compared with the 50-μl, but not the 100-μl group. For the normalized samples, mean Cq was similar between the 50-μl and 100-μl groups, but the 200-μl group had a lower mean Cq compared with both other groups. **(B)** RT-qPCR from the spike-in UniSp6 revealed a similar RT efficiency for all samples. **(C)** Addition of the MS2 carrier RNA did not improve the miR-23a-3p yield. **(D)** With ddPCR setup 3, the 200-μl group did not have a higher mean miR-23a-3p copy number than either the 50-μl or 100-μl group. For the normalized samples, however, the expression pattern was similar to that of RT-qPCR. **(E)** Setup 6 in ddPCR performed better with the un-normalized samples, as it showed a higher mean miR-23a-3p copy number in the 200-μl group than in the 50-μl group, as with RT-qPCR. Further, it also performed better with the normalized samples, since it reduced the difference between the 200-μl and 50-μl groups to 1.86-fold (p < 0.05), whereas in setup 3, it was 2.37-fold (p < 0.01). As a result of normalization, the mean miR-23a-3p Cq was higher and the mean copy number was lower in the 100-μl group than in its corresponding un-normalized counterpart, but not in the other groups. Statistical significances: *p < 0.05 and **p < 0.01 compared with the 50-μl group within normalized or un-normalized conditions; ##p < 0.01 compared to the 100-μl group within normalized or un-normalized conditions; +p < 0.05 normalized group compared with the same volume from the un-normalized group. Abbreviations: Cq, Cycle quantification; ddPCR, Droplet digital PCR; RT-qPCR, Reverse transcriptase-quantitative polymerase chain reaction.

RT-qPCR from un-normalized RNA was also performed with the miRCURY chemistry. Similar to TaqMan chemistry, the mean miR-23a-3p Cq decreased with a corresponding increase in the starting plasma volumes, when the 100-μl and 200-μl groups were compared with the 50-μl group (p < 0.01 and p < 0.05 respectively). With this chemistry, however, the unnormalized 200-μl group had a high within-group SD, because of which the mean Cq in this group was not different from that in the 100-μl (p > 0.05) (Fig. 5A). Unlike TaqMan RT-qPCR where the mean Cq value correlated with the Qubit concentration only in the 50-μl group, miRCURY RT-qPCR revealed a correlation between mean Cq and Qubit concentrations for all the three plasma volume groups (p < 0.05 for all) (Supplementary Table S12).

As with TaqMan RT-qPCR from small RNA concentration-normalized samples, the 50-μl and 100-μl plasma volume groups had similar Cq values (p > 0.05). The 200-μl group still had lower Cq in comparison with the other groups (p < 0.01 for both). SD and CV% within each group were reduced for all the three plasma volume groups after normalization (Supplementary Table S15).

RT-qPCR for the spike-in control UniSp6 revealed similar Cq values for all un-normalized and normalized samples, indicating no variability in the cDNA synthesis reactions (Fig. 5B).

#### RNA extractions with carrier (setup phase)

Because Qubit did not reliably measure the small RNA concentrations for the carrier-added samples, miRNA quantification for these were performed with TaqMan and miRCURY RT-qPCR from un-normalized samples only. Addition of the carrier RNA did not improve miR-23a-3p detection in any of the two chemistries. Rather, mean miR-23a-3p Cq for the carrier-added elutions was slightly higher than that of the no-carrier samples (p < 0.05 for both chemistries, Figs. 4C and 5C). Because no improvement in the miR-23a-3p yield was observed in RT-qPCR, we did not proceed to ddPCR with these samples.

#### miRCURY chemistry for RNA extractions without carrier (validation phase)

**Figure 6:**
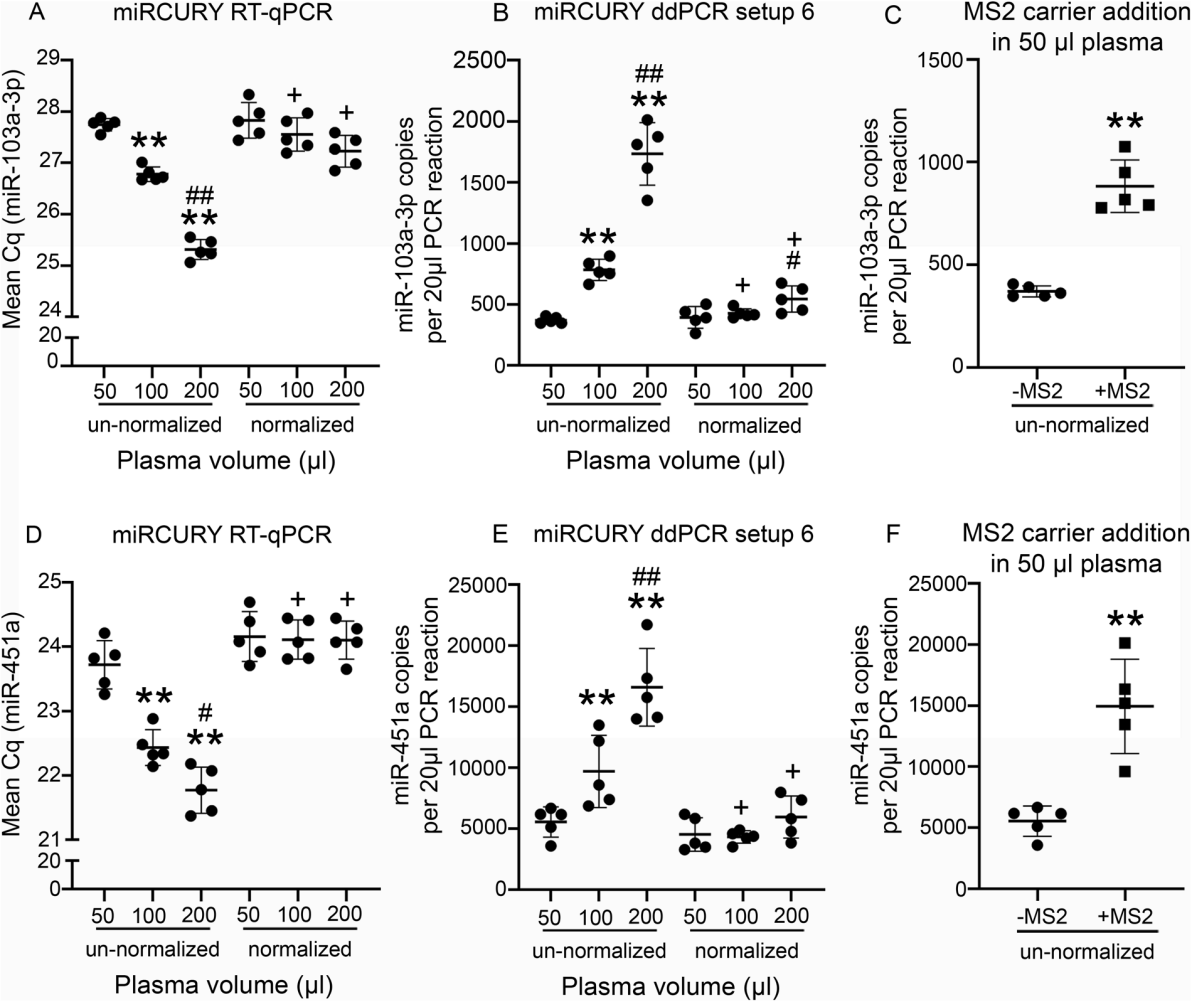
Concentration-normalization in validation phase revealed similar miR-103a-3p levels in the 50-μl and 100-μl groups, and similar miR-451a levels in all the three plasma volume groups. **(A,D)** As expected with un-normalized samples, mean miR-103a-3p and miR-451a Cq decreased with increase in starting plasma volumes. Unlike miR-23a-3p in the setup phase, miRCURY RT-qPCR from the normalized samples revealed similar miR-103a-3p and miR-451a levels in all the three groups. **(B,E)** With miRCURY ddPCR setup 6, un-normalized samples revealed increased miR-103a-3p and miR-451a copy numbers with increase in starting plasma volumes. For miR-103a-3p, the normalized 50-μl and 100-μl groups had similar levels, but the 200-μl group still showed higher miR-103a-3p levels than the 100-μl group. On the other hand, all the three groups showed normalized miR-451a copy number. **(C,F)** Addition of the MS2 carrier RNA improved both miR-103a-3p and miR-451a yield (miR-103a-3p: 2.38-fold, p < 0.01; miR-451a: 2.69-fold, p < 0.01). As a result of normalization, the mean miR-103a-3p and miR-451a Cq were higher and the mean copy numbers were lower in the 100-μl and 200-μl groups than their corresponding un-normalized counterparts. Statistical significances: **p < 0.01 compared with the 50-μl group within normalized or un-normalized conditions; ^#^p < 0.05 and ^##^p < 0.01 compared to the 100-μl group within normalized or un-normalized conditions; +p < 0.05 normalized group compared with the same volume from the un-normalized group. Abbreviations: Cq, Cycle quantification; ddPCR, Droplet digital PCR; RT-qPCR, Reverse transcriptase-quantitative polymerase chain reaction.

As expected from the setup phase, mean miR-103a-3p and miR-451a Cq decreased with corresponding increase in starting plasma volumes (Fig. 6A,D). However, the mean Cq values did not correlate with Qubit small RNA concentration measurements (p > 0.05; Supplementary Tables S13-S14). With small RNA concentration-normalization, both the normalized 100-μl and 200-μl plasma volume groups had similar Cq as the normalized 50-μl group (p > 0.05). However, SD and CV% within each group were small for un-normalized miR-103a-3p and miR-451a. Hence, with the normalization, no reduction in SD and CV% were observed except for miR-451a in the 200-μl plasma volume group (Supplementary Table S15).

### Comparison of miR-23a-3p, miR-103a-3p and miR-451a ddPCR from un-normalized and concentration-normalized RNA samples

#### TaqMan chemistry for RNA extractions without carrier (setup phase)

To obtain absolute copy numbers for plasma miR-23a-3p, we performed ddPCR. With un-normalized cDNA, the pattern was similar to that of RT-qPCR. Mean miR-23a-3p copy numbers for the 100-μl and 200-μl plasma volume groups were higher than that for the 50-μl group (p < 0.05 and p < 0.01 respectively). Similarly, the 200-μl group also had higher mean miR-23a-3p copy number than the 100-μl group (p < 0.01) (Fig. 4B**)**. In contrast to TaqMan RT-qPCR where the mean miR-23a-3p Cq correlated with the Qubit concentration only in the 50-μl plasma volume group, the mean miR-23a-3p copy number with TaqMan ddPCR correlated with Qubit concentrations only in the 100-μl group (p < 0.05, Supplementary Table S11).

Similar to TaqMan RT-qPCR, the mean miR-23a-3p copy number in the normalized 100-μl plasma volume group was not different from that in the normalized 50-μl group (p > 0.05), but the normalized 200-μl group still had a higher copy number than either of the other plasma volume groups (p < 0.01 for both). SD within each group reduced for all the three plasma volumes whereas CV% reduced only for the 50-µl and 100-μl (Supplementary Table S15).

Mean miR-23a-3p Cq and the mean copy number from TaqMan RT-qPCR and ddPCR correlated only for the un-normalized 50-μl group (p < 0.05, Supplementary Table S11). For normalized samples, there was correlation only for the 200-μl group (p < 0.01, Supplementary Table S11).

#### miRCURY chemistry for RNA extractions without carrier (setup phase)

Among the multiple optimization conditions tested for miRCURY miR-23a-3p ddPCR with un-normalized cDNA, setups 3 and 6 (Supplementary Table S10) performed best for the 50-μl plasma volume group. This was based on the correlation of the mean miR-23a-3p copy number with both the Qubit concentrations (p < 0.05 for both setups), and the mean miR-23a-3p Cq values (p < 0.01 for both setups) (Supplementary Table S12). For the un-normalized samples, the ddPCR pattern with setup 6 was more similar to that of RT-qPCR, in comparison with setup 3 (Fig. 5E). With setup 3, the mean miR-23a-3p copy number in the 200-μl group was not higher than that in either the 50-μl or 100-μl group (p > 0.05 for both) (Fig. 5D).

We routinely perform miRNA biomarker analysis from 50 μl of plasma^19^. Because setups 3 and 6 performed most optimally for un-normalized cDNA in the 50-μl group, we selected those setups for running the normalized cDNA samples. Setup 6 also performed better with the normalized samples than setup 3, as it reduced the difference between the normalized 50-μl and 200-μl groups (1.86-fold, p < 0.05 in setup 6 compared to 2.37-fold, p < 0.01 in setup 3, Fig. 5D,E). Reduced within group SD and CV% was observed as a result of normalization for all three plasma volumes, and with both setups (Supplementary Table S15).

When correlations between the mean miR-23a-3p Cq and the mean copy number from RT-qPCR and ddPCR chemistries was evaluated for the normalized samples, they correlated only for the 100-μl group with setup 3 (p < 0.05), but not with setup 6 (p > 0.05) (Supplementary Table S12).

#### miRCURY chemistry for RNA extractions without carrier (validation phase)

For the un-normalized samples, miR-103a-3p and miR-451a expression patterns with ddPCR were similar to that of RT-qPCR. Mean miR-103a-3p and miR-451a copy numbers increased with increase in starting plasma volumes (Fig. 6B,E). However, mean miR-103a-3p copy number did not correlate with Qubit concentrations (p > 0.05, Supplementary Table S13). For miR-451a, mean copy number correlated negatively with Qubit concentrations for the 50-μl plasma volume group, instead of the expected positive correlation (p < 0.05, Supplementary Table S14). With small RNA concentration normalization, the 100-μl plasma volume group had similar mean miR-103a-3p and miR-451a copy numbers as the 50-μl group. The 200-μl group also had similar miR-451a copies as the 50-μl and 100-μl. However, the 200-μl group still had higher miR-103a-3p copies in comparison to the 100-μl. As a result of normalization, SD within group reduced for the 100-μl and 200-μl plasma volume groups, but CV% reduced only for the 100-μl plasma volume group (Supplementary Table S15).

There was no correlation between mean miR-103a-3p Cq and copy numbers from miRCURY RT-qPCR and ddPCR for the un-normalized or normalized samples (p > 0.05; Supplementary Table S13). For miR-451a, there was correlation between mean Cq and copy number for the un-normalized samples in the 200-μl plasma volume group (p < 0.05; Supplementary Table S14). For the normalized samples, there was correlation only for 50-μl (p < 0.05; Supplementary Table S14).

#### miRCURY chemistry for RNA extractions with carrier (validation phase)

In the validation phase, miR-103a-3p and miR-451a levels were analysed from the un-normalized carrier-added samples with miRCURY ddPCR. miR-103a-3p and miR-451a copies were higher in the carrier-added samples compared to the ones without carrier (miR-103a-3p: 2.38-fold, p < 0.01; miR-451a: 2.69-fold, p < 0.01; (Fig. 6C,F).

In the setup phase, we had observed higher mean miR-23a-3p Cq levels with RT-qPCR in the carrier-added samples in comparison to the ones without carrier, but ddPCR was not performed. Thus, in the validation phase, we re-analyzed miR-23a-3p levels from the samples with and without carrier with ddPCR. Unlike RT-qPCR, no difference in mean miR-23a-3p copy number was observed between the two groups with ddPCR (1.01-fold, p > 0.05; Supplementary Fig. S5).

### Comparison of the a-priori small RNA concentration normalization with posteriori normalization

Adjusting the small RNA concentrations of the RNA samples prior to input to RT reaction resulted in an a-priori normalization. We also compared this with a posteriori normalization, where the miR-23a-3p, miR-103a-3p and miR-451a copies from un-normalized TaqMan and/or miRCURY ddPCR were normalized by the small RNA concentrations from Qubit using the following formula:

$$\frac{miRNA\,copies\,per\,20\,\mu l\,PCR\,reaction\,from\,un-normalized\,ddPCR}{small\,RNA\,concentration\,measured\,by\,Qubit}$$

With the posteriori normalization, miR-23a-3p and miR-451a revealed similar concentration across all the three plasma volume groups. However, for miR-103a-3p, the 200-μl plasma volume group had higher concentration than both the 50-μl and 100-μl groups (Supplementary Fig. S6). SD within each plasma volume group from posteriori normalization was not comparable to the un-normalized condition, since we obtained a normalized value with posteriori method instead of absolute copy numbers. However, CV% within group for miR-23a-3p reduced in the 50-µl 100-µl plasma volumes with TaqMan ddPCR, and in all the three groups with miRCURY ddPCR. For miR-103a-3p CV% within group reduced only in 200 μl. For miR-451a CV% within group reduced only in 100 μl (Supplementary Table S15).

## Discussion

Circulating plasma miRNAs are currently attracting great interest as minimally invasive biomarkers of various diseases. Their quantification is complicated by several pre-analytical and technical factors, however, both in the pre-clinical and clinical settings. In this work, we addressed some of these factors in the pre-clinical setting with rodent plasma. We had four main findings. First, spectrophotometric analysis of plasma hemolysis is crucial, particularly if the plasma is pipetted from the vicinity of the erythrocyte layer. Second, the Qubit miRNA assay can be used to measure eluted small RNA concentrations from rat plasma if carrier RNA is not added during the RNA extraction process. Third, addition of a carrier RNA during RNA extraction enhances extraction of only selected miRNAs. Fourth, small RNA concentration normalization followed by miRNA quantification with ddPCR can be performed to reduce the effect of sample-to-sample variations in RNA concentrations occuring during RNA extraction.

We identified that plasma aliquots pipetted from the vicinity of the erythrocyte layer had a higher proportion of hemolysis. Several methods for detecting hemolysis in human serum/plasma samples have been published^13,16,17^. For rat tail-vein plasma samples, we previously identified that hemoglobin absorbance measurement with the NanoDrop at 414 nm is more sensitive than the dCt(miR-23a – miR-451a) miRNA ratio or visual inspection (414 nm at spectrophotometer > miRNA ratio > visual inspection)^18^. In that study, we concluded that similar to human plasma^13,22^, absorbance > 0.20–0.25 at 414 nm is also indicative of hemolysis in rodents. In the present study, we investigated the pattern of rat tail-vein plasma hemolysis in further detail. Although NanoDrop measurements revealed that plasma from the individual EDTA-A or the EDTA-B tubes were not hemolysed, aliquoting revealed that the later aliquots were more hemolysed than the first ones. This was because the hemolysis values of the individual aliquots were affected by their vicinity to the erythrocyte layer. As a result, hemolysis values measured from the EDTA tubes and the individual plasma aliquots mostly did not correlate. This indicated that manual pipetting of plasma increases the risk of contamination, which is why automated pipettes are currently gaining popularity in clinical settings^35^. When comparing hemolysis values of individual aliquot tubes measured NanoDrop-1000 and Denovix DS-11 spectrophotometer, we identified comparable performance of the machines.

For our rat tail-vein plasma samples, the Qubit miRNA assay detected an increase in small RNA concentrations with an increase in the starting plasma volumes. The Qubit fluorometer has already been identified as a reliable platform for quantification of RNA from tissue^36^, as well as circulating DNA^37^ and total RNA^38,39^. Superior performance has also been reported for the Qubit miRNA assay in quantifying circulating miRNA concentration in human plasma samples, compared with spectrophotometric methods^14^. However, the previous study also highlighted that the Qubit miRNA assay detects all forms of small RNAs and are not specific to miRNAs. In the present study, we evaluated the Qubit miRNA assay for our rat plasma samples. We performed the measurements with two different pipettes and on two separate days, involving one freeze-thaw cycle of the RNA. With both pipettes and measurements, an increase in the small RNA concentration was observed with an increase in starting plasma volume. Importantly, there was no difference between the two measurements on successive days, indicating that the small RNA concentrations were unaffected by one freeze-thaw cycle. Qubit revealed a mean CV of 11% when the two pipettes and measurements were compared, which is consistent with the observations in the human plasma study^14^. The 5-μl pipette did not reduce the CV% in comparison with the 10-μl pipette. However, the 200-µl plasma volume group showed lower CV% compared to the 50-µl, with significant correlations when the different measurements and pipettes were compared. This indicates that the Qubit measurements were more consistent for RNA extracted from a larger plasma volume, given it is within the limit of saturation. Good performance of Qubit from 200-µl plasma has also been observed in another recent study^40^.

Addition of a carrier RNA during RNA extraction from plasma increases miRNA recovery^41^, but reports have been contradictory regarding the performances of MS2, yeast RNA, or glycogen as carriers^24,42,43^. Further, these studies have been performed in human plasma samples, and Qubit was not used to measure eluted RNA concentrations. With our rodent plasma samples, Qubit detected a very high small RNA concentration when extractions were performed in presence of the MS2 carrier. Ramon-Nunez *et al*. had also observed highest total RNA concentration with addition of MS2 carrier, but based on electropherogram analysis, they concluded yeast RNA to be a better carrier for small RNA and miRNA extraction than MS2^24^. Importantly, we also observed a very high small RNA concentration with Qubit even from the no-template control where RNA extraction was performed from a water sample with MS2 carrier addition. This finding revealed that the carrier RNA gets eluted when the miRNeasy columns are used. Further, even though its size is larger than that of the small RNAs, the Qubit miRNA assay detects it. Thus, isolated RNA concentrations get masked with the use of MS2 carrier^24^.

Further, while most studies emphasize the fact that carrier RNA addition improves miRNA recovery from serum/plasma samples, it is also highlighted that the recovery depends on the relative abundance of the miRNA in the sample (SRA), ΔG and GC content^24^. In the setup phase MS2 carrier-added samples showed higher mean miR-23a-3p Cq levels than the samples without carrier, whereas in the validation phase, no difference in miR-23a-3p copy numbers was observed with ddPCR. This indicates a possible inhibition of RT-qPCR due to the co-extracted carrier RNA which is resolved with the more tolerant ddPCR method^44,45^. However, ddPCR revealed that the levels for miR-103a-3p and miR-451a doubled with carrier addition, whereas there was no improvement in miR-23a-3p yield, indicating that the addition of MS2 carrier RNA impacts the extraction of miRNAs differently.

A limited number of analyses of miRNAs from rat plasma have been performed. Two of these studies used 200 μl plasma for RNA extraction^46,47^. According to Van Vliet et al^18^., a 50–200 μl plasma volume is suitable for miRNA analysis from rodents. In our study, we observed an increase in the concentrations of the analysed miRNAs with increase in starting plasma volumes. Thus, our findings confirm that 50–200 μl of starting plasma volume is suitable for rodents, with no significant PCR inhibition.

The major advantage of ddPCR over RT-qPCR is its reduced sensitivity to PCR inhibitors and its ability to perform absolute quantification, thereby eliminating the need for endogenous references or standard calibrator curves^25,44,48^. In comparison with RT-qPCR, most studies have also demonstrated similar or improved sensitivity, precision, and reproducibility of ddPCR^25,44,49^, with very good correlation between the two techniques^25,50,51^. Studies comparing the TaqMan and miRCURY chemistries have revealed similar performance of both chemistries for abundantly expressed miRNAs^52,53^. For low-abundance miRNAs, however, miRCURY is preferred over TaqMan^53^, although some variability in the measurements has been observed^52^. With ddPCR, performance of both chemistries is comparable^32^. In our study, performance of the TaqMan and miRCURY chemistries were comparable for the un-normalized and the concentration-normalized samples with RT-qPCR and ddPCR.

Total RNA content as a normalizer for miRNA RT-qPCR data was previously studied in tissue samples, but did not perform well^54^. For circulating miRNA analysis, a fixed RNA sample volume is typically used for cDNA synthesis due to poor estimation of the eluted RNA concentrations^26,27^. In this study, we attempted to normalize the plasma miR-23a-3p, miR-103a-3p and miR-451a levels of the individual samples to the total small RNA content based on Qubit assay detection. With this normalization, similar miRNA concentration could be achieved from different starting plasma volumes. The 100-μl plasma volume group had similar miRNA concentration as the 50-μl for all the three tested miRNAs (miR-23a-3p, miR-103a-3p and miR-451a). The 200-μl group also had similar miR-451a concentration as in 50-µl and 100-µl, but for miR-23a-3p and miR-103a-3p it showed a trend towards normalization. Since these variations were observed with both PCR chemistries, it indicated that normalization performed based on the total small RNA concentration measurement from Qubit does not perfectly normalize miRNA concentrations when 200 μl plasma volume is used as a starting material.

We also compared our a-priori normalization method with a posteriori normalization, wherein miRNA levels from the un-normalized samples were normalized to the Qubit concentration. Overall, the a-priori and posterior normalizations resulted in similar miRNA expression patterns. However, since ddPCR based on a-priori normalization yields absolute miRNA copy numbers, this method may be preferred over the posteriori to generate cut-off thresholds for circulating miRNA biomarker analysis.

Within each starting plasma volume group, the normalized samples mostly had a lower CV% for miR-23a-3p in comparison to the un-normalized condition. However, reduction in CV% as a result of normalization was not that prominent for miR-103a-3p or miR-451a, both with a-priori and posteriori methods. This plausibly indicates that the total small RNA concentration-based normalization affects individual miRNA concentrations differently. Nevertheless, performing the small RNA concentration based normalization and comparing it to the un-normalized condition is crucial for individual miRNA candidate, particularly in settings where RNA isolation has to be performed from different starting plasma volumes (e.g., in clinical settings with variable sample volume availability), and where variations in RNA extraction efficiencies are observed.

### Methodologic limitations

Hemolysis measurements from the plasma samples were performed only with the NanoDrop-1000 and Denovix DS-11, as these were the only two spectrophotometers available to us. Thus, performance of the Denovix DS-11 in comparison with other spectrophotometers, e.g., the NanoDrop-2000, was not evaluated. For TaqMan, the standard chemistry with miRNA specific cDNA synthesis was only analysed. The recently introduced TaqMan advanced miRNA assays that implement the universal cDNA synthesis similar to miRCURY chemistry was not evaluated. Further, the TaqMan and miRCURY primer assays are designed for RT-qPCR systems, making it essential to evaluate their performances in ddPCR for each miRNA assay. We recommend testing and optimizing the chemistries and the normalization strategies described here for every target miRNA.

## Conclusion

Our data suggest that spectrophotometry-based detection of hemolysis in rodent plasma samples should be performed to select good quality plasma for miRNA biomarker analysis. The Qubit performs well in measuring small RNA concentrations from low abundance samples like the plasma. Further, starting from a fixed volume of non-hemolysed rodent plasma, we can apply the small RNA concentration-normalization approach with absolute quantification in ddPCR for plasma miRNA biomarker analysis.

## Supplementary information

Supplementary information.

## Data Availability

The datasets used and/or analyzed in the current study are available from the corresponding author on reasonable request.
