# Supplementary material for: Droplet digital polymerase chain reaction-based quantification of circulating microRNAs using small RNA concentration normalization
Source: Sci Rep. 2020 Jun 2;10:9012. doi: 10.1038/s41598-020-66072-z (PMC7265372; doi:10.1038/s41598-020-66072-z)
Supplement: Supplementary file 1 — Supplementary information. [file 41598_2020_66072_MOESM1_ESM.pdf]

Droplet digital polymerase chain reaction-based quantification of circulating microRNAs using small RNA concentration normalization

Shalini Das Gupta[1], Xavier Ekolle Ndode-Ekane[1], Noora Puhakka[1#*], Asla Pitkänen[1#]

[1]*A. I. Virtanen Institute for Molecular Sciences, University of Eastern Finland, PO Box 1627, FI-70211 Kuopio, Finland*

# shared last authorship

***Corresponding author:** Noora Puhakka, PhD, A. I. Virtanen Institute for Molecular Sciences, University of Eastern Finland, PO Box 1627, FI-70211 Kuopio, Finland, Tel: +358-40-355 3670, E-mail: noora.puhakka@uef.fi

Supplementary Table S1. Plasma hemolysis values in the EDTA-A and EDTA-B tubes immediately after centrifugation.

EDTA tube no.	NanoDrop Hemolysis value	EDTA tube no.	NanoDrop Hemolysis value
**48A**	0.14	**48B**	0.09
**49A**	0.08	**49B**	0.15
**50A**	0.07	**50B**	0.09
**51A**	0.09	**51B**	0.10
**52A**	0.10	**52B**	0.14
**53A**	0.16	**53B**	0.09
**54A**	0.14	**54B**	0.12
**55A**	0.18	**55B**	0.13
**56A**	0.15	**56B**	0.10
**57A**	0.13	**57B**	0.14
**58A**	0.14	**58B**	0.11
**59A**	0.10	**59B**	0.10
**60A**	0.14	**60B**	0.14
**61A**	0.15	**61B**	0.10
**62A**	0.09	**62B**	0.13
**63A**	0.28	**63B**	0.14
**64A**	0.16	**64B**	0.11
**65A**	0.11	**65B**	0.13
**66A**	0.10	**66B**	0.9
**67A**	0.07	**67B**	0.10
**68A**	0.14	**68B**	0.12
**69A**	0.10	**69B**	0.10
**70A**	0.14	**70B**	0.21
**71A**	0.11	**71B**	0.09
**72A**	0.08	**72B**	0.13
**73A**	0.11	**73B**	0.10
**74A**	0.10	**74B**	0.11
**75A**	0.09	**75B**	0.12
**76A**	0.15	**76B**	0.16
**77A**	0.11	**77B**	0.13

Abbreviations: Hemolysed samples are highlighted in red.

Supplementary Table S2. Plasma hemolysis values in the A3 aliquots of the EDTA-A tubes (used for method setup).

Aliquot tube no.	NanoDrop Hemolysis value	Denovix Hemolysis value	Visual detection
**48A3**	0.13	1.32	
**49A3**	0.13	1.26	
**50A3**	0.16	1.53	
**51A3**	0.18	2.04	
**52A3**	0.18	1.88	
**53A3**	0.12	1.25	
**54A3**	0.15	1.43	
**55A3**	0.21	2.06	
**56A3**	0.14	1.71	
**57A3**	0.17	1.55	
**58A3**	0.13	1.27	
**59A3**	0.11	1.11	
**60A3**	0.16	1.62	
**61A3**	0.11	1.07	
**62A3**	0.10	0.97	
**63A3**	0.54[L]	4.10[L]	Yes
**64A3**	0.11	1.06	
**65A3**	0.14	1.33	
**66A3**	0.10	0.99	
**67A3**	0.15	1.54	

Abbreviations: Aliquots detected as hemolysed by both NanoDrop and Denovix are highlighted in red. Visual detection indicates if the experienced researcher noted the aliquot as hemolyzed while pipetting. L: last aliquot of the EDTA-A tube due to smaller plasma volume.

Supplementary Table S3. Plasma hemolysis values in the A4 aliquots of the EDTA-A tubes (used for method setup).

Aliquot tube no.	NanoDrop Hemolysis value	Denovix Hemolysis value	Visual detection
**48A4**	0.13	1.49	
**49A4**	0.16	1.68	
**50A4**	0.12	1.23	
**51A4**	0.35[L]	3.84[L]	No
**52A4**	0.28	2.82	No
**53A4**	0.20	2.19	
**54A4**	0.16	1.69	
**55A4**	0.27[L]	2.52[L]	No
**56A4**	0.15	1.62	
**57A4**	0.20	1.94	
**58A4**	0.12	1.30	
**59A4**	0.21	2.19	
**60A4**	0.19	1.95	
**61A4**	0.12	1.27	
**62A4**	0.11	1.15	
**64A4**	0.16	1.52	
**65A4**	0.16	1.71	
**67A4**	0.32	3.26	No
**68A4**	0.19	1.99	
**69A4**	0.12	1.23	
**71A4**	0.12	1.35	
**72A4**	0.10	1.09	
**74A4**	0.13	1.37	
**75A4**	0.36[L]	3.69[L]	Yes
**76A4**	0.49[L]	5.17[L]	Yes
**77A4**	0.13	1.55	

Abbreviations: Aliquots detected as hemolysed by both NanoDrop and Denovix are highlighted in red. Visual detection indicates if the experienced researcher noted the aliquot as hemolyzed while pipetting. L: last aliquot of the EDTA-A tube due to smaller plasma volume.

Supplementary Table S4. Plasma hemolysis values in the A5 aliquots of the EDTA-A tubes (used for method setup).

Aliquot tube no.	NanoDrop Hemolysis value	Denovix Hemolysis value	Visual detection
**48A5**	0.20	2.47	
**49A5**	0.31	2.63	No
**50A5**	0.25	2.57#	No
**52A5**	0.40	4.40	No
**54A5**	0.18	1.63	
**56A5**	0.18	1.74	
**57A5**	0.29	3.54	No
**58A5**	0.19	1.81	
**60A5**	0.22	2.13	
**61A5**	0.16	1.53	
**62A5**	0.44	4.33	Yes
**64A5**	0.35	5.37	Yes
**65A5**	0.17	1.81	
**66A5**	0.17	1.83	
**67A5**	0.75	7.13	Yes
**68A5**	0.22	2.30	
**69A5**	0.20	2.19	
**70A5**	0.37	2.65	No
**71A5**	0.25	1.90	No
**72A5**	0.54	4.40	No
**73A5**	0.15	1.66	
**74A5**	0.14	1.50	
**77A5**	0.33	2.77	No

Abbreviations: Aliquots detected as hemolysed by both NanoDrop and Denovix are highlighted in red. # detected as hemolysed only by Denovix. Visual detection indicates if the experienced researcher noted the aliquot as hemolyzed while pipetting.

Supplementary Table S5. Plasma hemolysis values in the B4 aliquots of the EDTA-B tubes (used for method setup).

Aliquot tube no.	NanoDrop Hemolysis value	Denovix Hemolysis value	Visual detection
**48B4**	0.14	1.46	
**49B4**	1.05	6.43	No
**50B4**	0.13	1.24	
**51B4**	0.23	2.50	
**52B4**	0.31	3.25	No
**54B4**	0.26	2.73	No
**55B4**	0.18	1.73	
**56B4**	0.15	1.59	
**57B4**	0.22	2.27	
**58B4**	0.55	5.23	Yes
**59B4**	0.32	3.02	Yes
**60B4**	0.32	3.01	No
**61B4**	0.11	1.05	
**62B4**	0.16	1.55	
**63B4**	0.17	1.91	
**65B4**	0.52	3.93	Yes
**66B4**	0.14	1.35	
**67B4**	0.34	3.56	No
**68B4**	0.17	1.81	
**69B4**	0.11	1.18	
**70B4**	0.66	6.41	Yes
**71B4**	0.32*	2.37	No
**72B4**	0.35	3.74	No
**73B4**	0.42	2.93	Yes
**74B4**	0.20	3.72#	Yes
**76B4**	0.26*	2.18	No
**77B4**	0.54	3.97	Yes

Abbreviations: Aliquots detected as hemolysed by both NanoDrop and Denovix are highlighted in red. * detected as hemolysed only by NanoDrop, # detected as hemolysed only by Denovix. Visual detection indicates if the experienced researcher noted the aliquot as hemolyzed while pipetting.

Supplementary Table S6. Plasma hemolysis values in the A2 aliquots of the EDTA-A tubes (used for method validation).

Aliquot tube no.	NanoDrop Hemolysis value	Denovix Hemolysis value	Visual detection
**48A2**	0.12	1.28	
**49A2**	0.14	1.32	
**50A2**	0.11	1.03	
**51A2**	0.19	1.94	
**52A2**	0.16	1.73	
**53A2**	0.16	1.72	
**54A2**	0.15	1.41	
**55A2**	0.20	1.92	
**56A2**	0.15	1.32	
**57A2**	0.14	1.39	
**58A2**	0.12	1.17	
**59A2**	0.11	1.04	
**60A2**	0.16	1.65	
**61A2**	0.13	1.30	
**62A2**	0.10	0.95	
**63A2**	0.34	3.76	Yes
**64A2**	0.12	1.11	
**65A2**	0.13	1.33	
**66A2**	0.10	1.05	
**67A2**	0.15	1.40	

Abbreviations: Aliquots detected as hemolysed by both NanoDrop and Denovix are highlighted in red. Visual detection indicates if the experienced researcher noted the aliquot as hemolyzed while pipetting.

Supplementary Table S7. Plasma hemolysis values in the A3 aliquots of the EDTA-A tubes (used for method validation).

Aliquot tube no.	NanoDrop Hemolysis value	Denovix Hemolysis value	Visual detection
**68A3**	0.16	1.33	
**69A3**	0.10	0.78	
**70A3**	0.22	1.54	
**71A3**	0.12	0.93	
**72A3**	0.10	0.79	
**73A3**	0.12	1.07	
**74A3**	0.12	1.11	
**75A3**	0.17	1.38	
**76A3**	0.16	1.48	
**77A3**	0.12	1.07	

Supplementary Table S8. Plasma hemolysis values in the B3 aliquots of the EDTA-B tubes (used for method validation).

Aliquot tube no.	NanoDrop Hemolysis value	Denovix Hemolysis value	Visual detection
**48B3**	0.10	1.09	
**49B3**	0.10	1.01	
**50B3**	0.09	0.94	
**51B3**	0.12	1.16	
**52B3**	0.19	1.81	
**53B2**	0.10	0.95	
**54B3**	0.16	1.41	
**55B3**	0.15	1.32	
**56B3**	0.11	0.85	
**57B3**	0.15	1.21	
**58B3**	0.09	0.82	
**59B3**	0.16	1.35	
**60B3**	0.19	1.81	
**61B3**	0.10	0.84	
**62B3**	0.13	1.23	
**63B3**	0.11	1.07	
**64B3**	0.55[L]	5.51[L]	Yes
**65B3**	0.40*	2.46	Yes
**66B3**	0.09	0.90	
**67B3**	0.14	1.25	
**68B3**	0.13	1.18	
**69B3**	0.10	0.88	
**70B3**	0.28*	2.50	No
**71B3**	0.11	1.07	
**72B3**	0.26*	1.88	No
**73B3**	0.17	1.18	
**74B3**	0.13	1.40	
**75B3**	0.20	1.68	Yes
**76B3**	0.23	2.07	
**77B3**	0.17	1.34	

Abbreviations: Aliquots detected as hemolysed by both NanoDrop and Denovix are highlighted in red. * detected as hemolysed only by NanoDrop. Visual detection indicates if the experienced researcher noted the aliquot as hemolyzed while pipetting. L: last aliquot of the EDTA-A tube due to smaller plasma volume.

Supplementary Table S9. Correlation analysis between hemolysis values measured from the EDTA tubes (before freezing) and the individual plasma aliquots (after thawing) with NanoDrop-1000.

**A. Hemolysis in EDTA-A tube vs. aliquot tubes:**	
**Aliquot tubes**	**ρ**
A2	0.4
A3	0.2
A4	0.1
A5	-0.3
**B. Hemolysis in EDTA-B tube vs. aliquot tubes:**	
**Plasma volume**	**ρ**
B3	0.6**
B4	0.4*

Abbreviations: *p<0.05, **p<0.01

Supplementary Table S10. PCR reaction setups and thermal cycling conditions tested for TaqMan and miRCURY RT-qPCR and ddPCR at the setup phase.

**TaqMan chemistry**	
**Reaction setup for RT-qPCR (20 µl)**	**Thermal cycling condition**
10 µl PCR Master Mix, 7.67 µl nuclease-free water, 1 µl Small RNA Assay and 1.33 µl of un-normalized or normalized cDNA	50°C for 2 min, 95°C for 10 min followed by 40 cycles of 95°C for 15 sec and 60°C for 1 min.
**Reaction setup for ddPCR (20 µl)**	**Thermal cycling condition**
10 µl ddPCR supermix for probes, 7.67 µl nuclease-free water, 1 µl Small RNA Assay and 1.33 µl of un-normalized or normalized cDNA	95°C for 10 min, 40 cycles of 95°C for 15 sec and 60°C for 1 min, a final step of 98°C for 10 min and hold at 4°C.
**miRCURY chemistry**	
**Reaction setup for RT-qPCR (10 µl)**	**Thermal cycling condition**
5 µl PCR master mix, 1 µl nuclease-free water, 1 µl of the Small RNA assay and 3 µl of 1:30 diluted un-normalized or normalized cDNA	95°C for 2 min and 40 cycles of 95°C for 10 sec and 56°C for 1 min
**Reaction setups for ddPCR (20 µl)**	**Thermal cycling conditions**
**Setup 1:** 10 µl ddPCR EvaGreen supermix, 2 µl nuclease-free water, 2 µl of the Small RNA assay and 6 µl of 1:30 diluted un-normalized or normalized cDNA	**For setups 1-3:** 95°C for 5 min, 40 cycles of 95°C for 30 sec and 56°C for 1 min, 4°C for 5 min, 90°C for 5 min and hold at 4°C
**Setup 2:** 10 µl ddPCR EvaGreen supermix, 2 µl nuclease-free water, 1 µl of the Small RNA assay and 7 µl of 1:30 diluted un-normalized or normalized cDNA	
**Setup 3:** 10 µl ddPCR EvaGreen supermix, 3 µl nuclease-free water, 1 µl of the Small RNA assay and 6 µl of 1:30 diluted un-normalized or normalized cDNA	

**Setup 4:** 10 µl ddPCR EvaGreen supermix, 2 µl nuclease-free water, 2 µl of the Small RNA assay and 6 µl of 1:30 diluted un-normalized or normalized cDNA	**For setups 4-6:** 95°C for 5 min, 40 cycles of 95°C for 30 sec and 58°C for 1 min, 4°C for 5 min, 90°C for 5 min and hold at 4°C
**Setup 5:** 10 µl ddPCR EvaGreen supermix, 3 µl nuclease-free water, 1 µl of the Small RNA assay and 6 µl of 1:30 diluted un-normalized or normalized cDNA	
**Setup 6:** 10 µl ddPCR EvaGreen supermix, 1 µl nuclease-free water, 1 µl of the Small RNA assay and 8 µl of 1:30 diluted un-normalized or normalized cDNA	

Supplementary Table S11. Correlations between Qubit concentrations and TaqMan RT-qPCR and ddPCR for miR-23a-3p.

**C. Un-normalized cDNA:** Qubit concentration vs. mean miR-23a-3p Cq	
**Plasma volume**	**ρ**
50 µl	-0.9*
100 µl	-0.7
200 µl	-0.4
**D. Un-normalized cDNA:** Qubit concentration vs. mean miR-23a-3p copies	
**Plasma volume**	**ρ**
50 µl	0.8
100 µl	0.9*
200 µl	-0.6
**E. Un-normalized cDNA:** mean miR-23a-3p Cq vs. mean miR-23a-3p copies	
**Plasma volume**	**ρ**
50 µl	-0.9*
100 µl	-0.6
200 µl	0.0
**F. Normalized cDNA:** mean miR-23a-3p Cq vs. mean miR-23a-3p copies	
**Plasma volume**	**ρ**
50 µl	-0.4
100 µl	-0.7
200 µl	-1.0**

Abbreviations: *p<0.05, **p<0.01

Supplementary Table S12. Correlations between Qubit concentrations and miRCURY RT-qPCR and ddPCR for miR-23a-3p.

**Un-normalized cDNA: miRCURY RT-qPCR**	
A. Qubit concentration vs. mean miR-23a-3p Cq	
**Plasma volume**	**ρ**
50 µl	-0.9*
100 µl	-0.9*
200 µl	-0.9*
**Un-normalized cDNA: miRCURY ddPCR <u>Setup 1</u>**	
B. Qubit concentration vs. mean miR-23a-3p copies	
**Plasma volume**	**ρ**
50 µl	0.7
100 µl	0.6
200 µl	0.9*
C. Mean miR-23a-3p Cq vs. mean miR-23a-3p copies	
**Plasma volume**	**ρ**
50 µl	-0.9*
100 µl	-0.7
200 µl	-1.0**
**Un-normalized cDNA: miRCURY ddPCR <u>Setup 2</u>**	
D. Qubit concentration vs. mean miR-23a-3p copies	
**Plasma volume**	**ρ**
50 µl	0.7
100 µl	0.7
200 µl	0.7
E. Mean miR-23a-3p Cq vs. mean miR-23a-3p copies	
**Plasma volume**	**ρ**
50 µl	-0.9*
100 µl	-0.9*
200 µl	-0.9*
**Un-normalized cDNA: miRCURY ddPCR <u>Setup 3</u>**	
F. Qubit concentration vs. mean miR-23a-3p copies	
**Plasma volume**	**ρ**
50 µl	0.9*
100 µl	0.6
200 µl	0.7
G. Mean miR-23a-3p Cq vs. mean miR-23a-3p copies	
**Plasma volume**	**ρ**

50 µl	-1.0**
100 µl	-0.7
200 µl	-0.9*
**Un-normalized cDNA: miRCURY ddPCR Setup 4**	
H. Qubit concentration vs. mean miR-23a-3p copies	
**Plasma volume**	**ρ**
50 µl	0.9*
100 µl	0.0
200 µl	0.7
I. Mean miR-23a-3p Cq vs. mean miR-23a-3p copies	
**Plasma volume**	**ρ**
50 µl	-0.8
100 µl	-0.2
200 µl	-0.9*
**Un-normalized cDNA: miRCURY ddPCR Setup 5**	
J. Qubit concentration vs. mean miR-23a-3p copies	
**Plasma volume**	**ρ**
50 µl	0.7
100 µl	0.9*
200 µl	0.9*
K. Mean miR-23a-3p Cq vs. mean miR-23a-3p copies	
**Plasma volume**	**ρ**
50 µl	-0.9*
100 µl	-1.0**
200 µl	-1.0**
**Un-normalized cDNA: miRCURY ddPCR Setup 6**	
L. Qubit concentration vs. mean miR-23a-3p copies	
**Plasma volume**	**ρ**
50 µl	0.9*
100 µl	0.7
200 µl	1.0**
M. Mean miR-23a-3p Cq vs. mean miR-23a-3p copies	
**Plasma volume**	**ρ**
50 µl	-1.0**
100 µl	-0.6
200 µl	-0.9*
**Normalized cDNA: miRCURY ddPCR Setup 3**	
N. Mean miR-23a-3p Cq vs. mean miR-23a-3p copies	
**Plasma volume**	**ρ**
50 µl	0.1
100 µl	-0.9*

200 µl	-0.3
**Normalized cDNA: miRCURY ddPCR Setup 6**	
O. Mean miR-23a-3p Cq vs. mean miR-23a-3p copies	
**Plasma volume**	**ρ**
50 µl	-0.7
100 µl	-0.5
200 µl	-0.7

Abbreviations: *p<0.05, **p<0.01

Supplementary Table S13. Correlations between Qubit concentrations and miRCURY RT-qPCR and ddPCR for miR-103a-3p.

A. **Un-normalized cDNA:** Qubit concentration vs. mean miR-103a-3p Cq	
**Plasma volume**	**ρ**
50 µl	-0.3
100 µl	-0.3
200 µl	0.8
B. **Un-normalized cDNA:** Qubit concentration vs. mean miR-103a-3p copies	
**Plasma volume**	**ρ**
50 µl	-0.6
100 µl	0.1
200 µl	0.3
C. **Un-normalized cDNA:** mean miR-103a-3p Cq vs. mean miR-103a-3p copies	
**Plasma volume**	**ρ**
50 µl	-0.4
100 µl	-0.4
200 µl	0.0
D. **Normalized cDNA:** mean miR-103a-3p Cq vs. mean miR-103a-3p copies	
**Plasma volume**	**ρ**
50 µl	-0.6
100 µl	0.2
200 µl	-0.6

Supplementary Table S14. Correlations between Qubit concentrations and miRCURY RT-qPCR and ddPCR for miR-451a.

A. **Un-normalized cDNA:** Qubit concentration vs. mean miR-451a Cq	
**Plasma volume**	**ρ**
50 µl	-0.3
100 µl	-0.5
200 µl	-0.3
B. **Un-normalized cDNA:** Qubit concentration vs. mean miR-451a copies	
**Plasma volume**	**ρ**
50 µl	-0.9*
100 µl	-0.1
200 µl	0.1
C. **Un-normalized cDNA:** mean miR-451a Cq vs. mean miR-451a copies	
**Plasma volume**	**ρ**
50 µl	0.1
100 µl	-0.2
200 µl	-0.9*
D. **Normalized cDNA:** mean miR-451a Cq vs. mean miR-451a copies	
**Plasma volume**	**ρ**
50 µl	-0.9*
100 µl	-0.6
200 µl	-0.3

Abbreviations: *$p<0.05$

Supplementary Table S15. Comparison of within group standard deviation (SD) and coefficient of variation (CV%) after a-priori and posteriori normalization to the un-normalized condition in ddPCR.

A. Setup phase: miR-23a-3p TaqMan RT-qPCR						
**Plasma volume**	**Un-normalized**		**A-priori normalized**		**Posteriori normalized**	
	**SD**	**CV%**	**SD**	**CV%**	**SD**	**CV%**
50 µl	0.31	1.29	0.19	0.77		
100 µl	0.23	0.97	0.16	0.66		
200 µl	0.08	0.34	0.19	0.80		

B. Setup phase: miR-23a-3p TaqMan ddPCR						
**Plasma volume**	**Un-normalized**		**A-priori normalized**		**Posteriori normalized**	
	**SD**	**CV%**	**SD**	**CV%**	**SD**	**CV%**
50 µl	1103.12	28.67	400.85	14.98	2.06	14.19
100 µl	1515.46	25.97	428.45	13.11	1.79	12.88
200 µl	1040.37	9.21	834.12	17.09	5.31	27.79

C. Setup phase: miR-23a-3p miRCURY RT-qPCR						
**Plasma volume**	**Un-normalized**		**A-priori normalized**		**Posteriori normalized**	
	**SD**	**CV%**	**SD**	**CV%**	**SD**	**CV%**
50 µl	0.30	1.42	0.15	0.69		
100 µl	0.29	1.40	0.16	0.74		
200 µl	1.05	5.25	0.30	1.45		

D. Setup phase: miR-23a-3p miRCURY ddPCR Setup 3						
**Plasma volume**	**Un-normalized**		**A-priori normalized**		**Posteriori normalized**	
	**SD**	**CV%**	**SD**	**CV%**	**SD**	**CV%**
50 µl	949.42	30.10	699.81	24.55	1.49	12.56
100 µl	1001.66	15.01	497.21	13.82	2.07	12.81
200 µl	7597.09	62.66	886.78	13.14	10.46	56.37

E. Setup phase: miR-23a-3p miRCURY ddPCR Setup 6						
Plasma volume	Un-normalized		A-priori normalized		Posteriori normalized	
	SD	CV%	SD	CV%	SD	CV%
50 µl	1471.42	28.44	799.42	27.12	2.28	11.68
100 µl	3065.22	27.55	354.02	10.61	3.88	14.74
200 µl	11107.93	59.95	1210.48	22.10	14.47	51.28

F. Validation phase: miR-103a-3p miRCURY RT-qPCR						
Plasma volume	Un-normalized		A-priori normalized		Posteriori normalized	
	SD	CV%	SD	CV%	SD	CV%
50 µl	0.12	0.44	0.35	1.25		
100 µl	0.14	0.53	0.33	1.19		
200 µl	0.19	0.77	0.31	1.14		

G. Validation phase: miR-103a-3p miRCURY ddPCR Setup 6						
Plasma volume	Un-normalized		A-priori normalized		Posteriori normalized	
	SD	CV%	SD	CV%	SD	CV%
50 µl	26.51	7.15	89.15	22.64	0.44	17.68
100 µl	87.73	11.20	37.69	8.82	0.40	12.65
200 µl	255.12	14.73	106.94	19.64	0.43	11.18

H. Validation phase: miR-451a miRCURY RT-qPCR						
Plasma volume	Un-normalized		A-priori normalized		Posteriori normalized	
	SD	CV%	SD	CV%	SD	CV%
50 µl	0.38	1.58	0.39	1.60		
100 µl	0.28	1.24	0.31	1.27		
200 µl	0.36	1.66	0.30	1.23		

I. Validation phase: miR-451a miRCURY ddPCR Setup 6						
Plasma volume	Un-normalized		A-priori normalized		Posteriori normalized	
	SD	CV%	SD	CV%	SD	CV%
50 µl	1237.76	22.30	1380.71	30.49	11.72	30.88
100 µl	2967.33	30.58	512.22	11.85	11.22	28.71

200 µl	3182.71	19.18	1724.28	28.94	7.59	20.54

Supplementary Table S16. Statistical comparisons performed in this study.

**Step 1: Hemolysis measurements**	
**Comparison**	**Statistical test used**
Between EDTA tubes and aliquots prepared from them (EDTA-A and A2, A3, A4, A5; EDTA-B and B3, B4)	Friedman's test followed by post-hoc Wilcoxon analysis
	Spearman's rho
Between individual aliquot tubes (setup: A3, A4, A5, B4; validation: A2, A3, B3)	Kruskal Wallis test followed by post-hoc Mann Whitney U test
NanoDrop vs. Denovix hemolysis measurements	Spearman's rho
**Step 2: Small RNA concentration measurement with Qubit**	
**Comparison**	**Statistical test used**
Small RNA concentrations between different plasma volume groups (50-µl, 100-µl and 200-µl)	Kruskal Wallis test followed by post-hoc Mann Whitney U test
Small RNA concentrations between different measurements (m1 and m2) for the same plasma volume group	Wilcoxon signed rank test
Coefficient of variation analysis between different plasma volume groups	Kruskal Wallis test followed by post-hoc Mann Whitney U test
Coefficient of variation analysis within same plasma volume groups	Wilcoxon signed rank test
Correlation between the small RNA concentrations measured by the 10-µl and 5-µl pipettes	Spearman's rho
Correlation between the small RNA concentrations measured over different days (m1 and m2) by each pipette	Spearman's rho
**Step 3: RT-qPCR and ddPCR analysis from un-normalized and concentration-normalized samples (TaqMan and miRCURY)**	
**Comparison**	**Statistical test used**
Between different plasma volume groups	Kruskal Wallis test followed by post-hoc Mann Whitney U test
Between un-normalized and normalized RNA from the same plasma volume group	Wilcoxon signed rank test
Between no-carrier and carrier added RNA	Mann Whitney U test
Between Qubit concentration and miRNA Cq from RT-qPCR	Spearman's rho
Between Qubit concentration and miRNA copies from ddPCR	Spearman's rho
Between miRNA Cq and copies from RT-qPCR and ddPCR	Spearman's rho

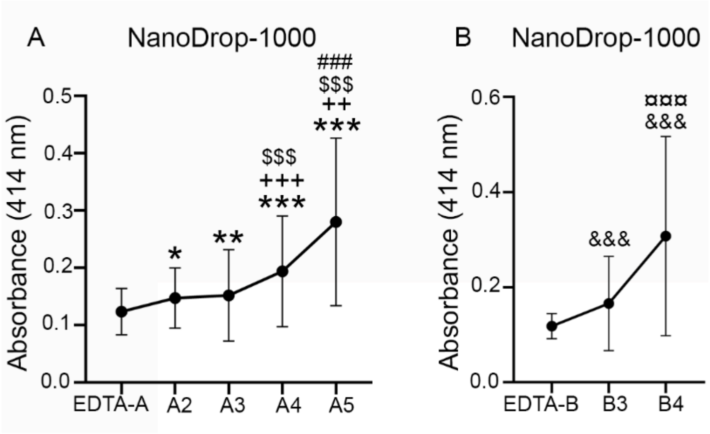

Supplementary figure 1. Comparison of plasma hemolysis values before and after freeze-thaw. (A-B) Plasma hemolysis values measured with NanoDrop-1000 from the EDTA-A and EDTA-B tubes immediately after centrifugation were compared with the hemolysis values measured from the individual aliquot tubes after melting. The aliquots used in both setup and validation phases were included in this comparison. Statistical significances: *p<0.05, **p<0.01 and ***p<0.001 compared to the EDTA-A tube, ++p<0.01 and +++p<0.001 compared to A2 aliquot tube, $$$p<0.001 compared to A3 aliquot tube, ###p<0.001 compared to A4 aliquot tube, &&&p<0.001 compared to EDTA-B tube, ¤¤¤p<0.001 compared to B3 aliquot tube. Abbreviations: A2, the 2nd 50-µl plasma aliquot from the EDTA-A tube; A3, the 3rd 50-µl plasma aliquot from the EDTA-A tube; A4, the 4th 50-µl plasma aliquot from the EDTA-A tube; A5, the 5th 50-µl plasma aliquot from the EDTA-A tube; B3, the 3rd 50-µl plasma aliquot from the EDTA-B tube; B4, the 4th 50-µl plasma aliquot from the EDTA-B tube; EDTA, Ethylenediaminetetraacetic acid.

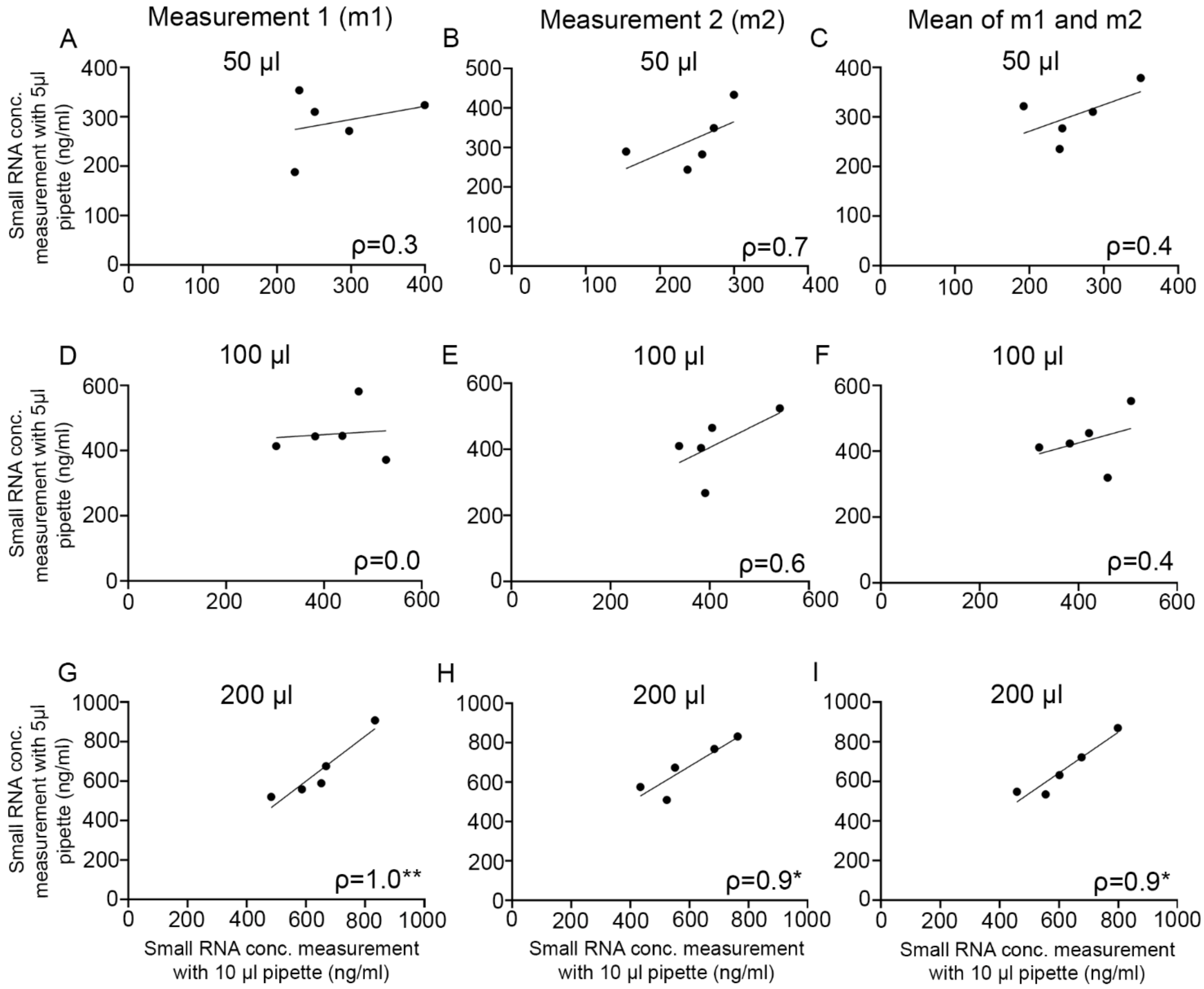

Supplementary figure 2. Effect of 10-µl and 5-µl pipette in pipetting the RNA eluate to the Qubit assay (setup phase). Small RNA concentrations were measured immediately after elution (m1) and on one other day following one freeze-thaw cycle (m2). For both measurements, 1 µl of RNA eluate was pipetted to the Qubit assay with either a 10-µl pipette or a 5-µl pipette. **(A-F)** For the 50-µl and 100-µl plasma volume groups, there was no correlation between the small RNA concentrations measured with the 10-µl and 5-µl pipettes in m1 or m2 or their mean ($p>0.05$). **(G-I)** In the 200-µl group, however, the small RNA concentrations measured by the two pipettes correlated both in m1 ($p<0.01$) and m2 ($p<0.05$), and also when the mean of the two measurements was analyzed ($p<0.05$). Statistical significances: *$p<0.05$, **$p<0.01$. Abbreviations: conc., Concentration.

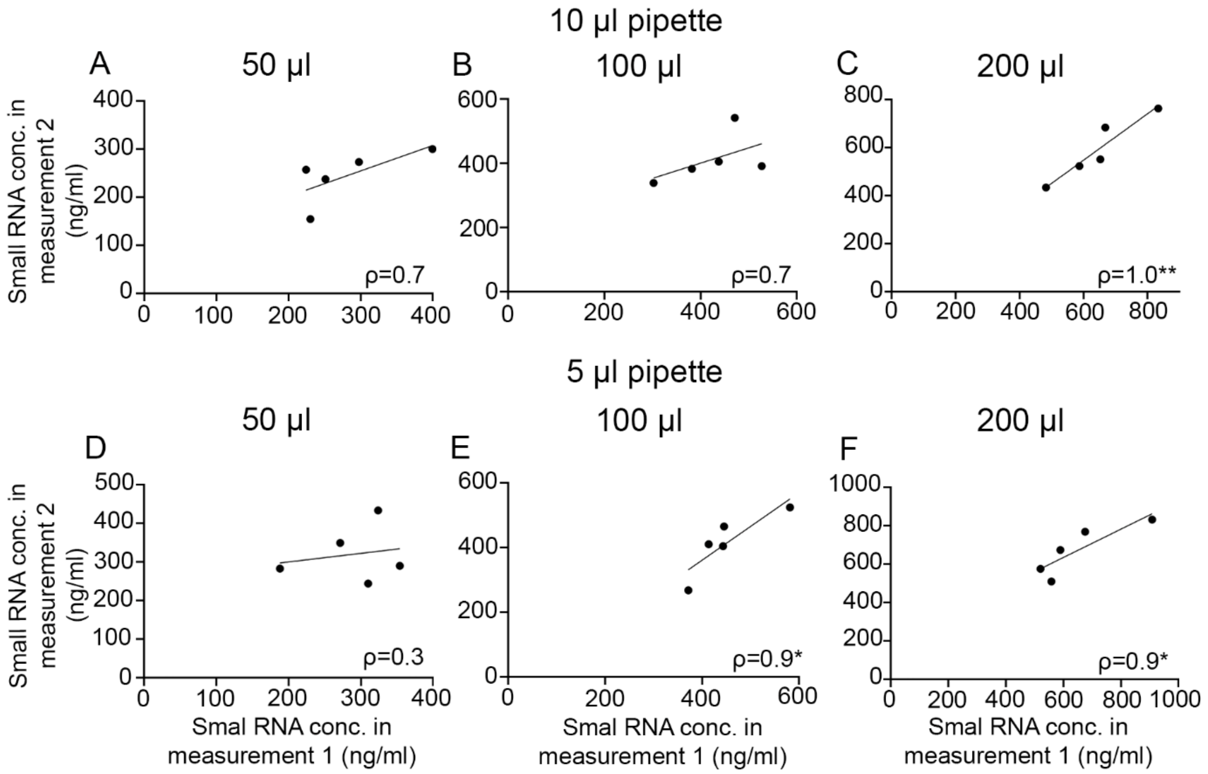

Supplementary figure 3. Correlation between small RNA concentration measurements m1 and m2 involving one RNA freeze-thaw cycle (setup phase). **(A, D)** There was no correlation between m1 (small RNA concentration measured immediately after elution) and m2 (small RNA concentration measured after one freeze-thaw cycle) for the 50-µl group, either with the 10-µl pipette or the 5-µl pipette ($p>0.05$). In the 100-µl group, m1 and m2 were correlated when using the **(E)** the 5-µl pipette ($p<0.05$), but not **(B)** the 10-µl pipette ($p>0.05$). For the 200-µl group, correlations were significant with **(C, F)** both pipettes ($p<0.01$ and $p<0.05$ respectively). Statistical significances: *$p<0.05$, **$p<0.01$. Abbreviations: conc., Concentration.

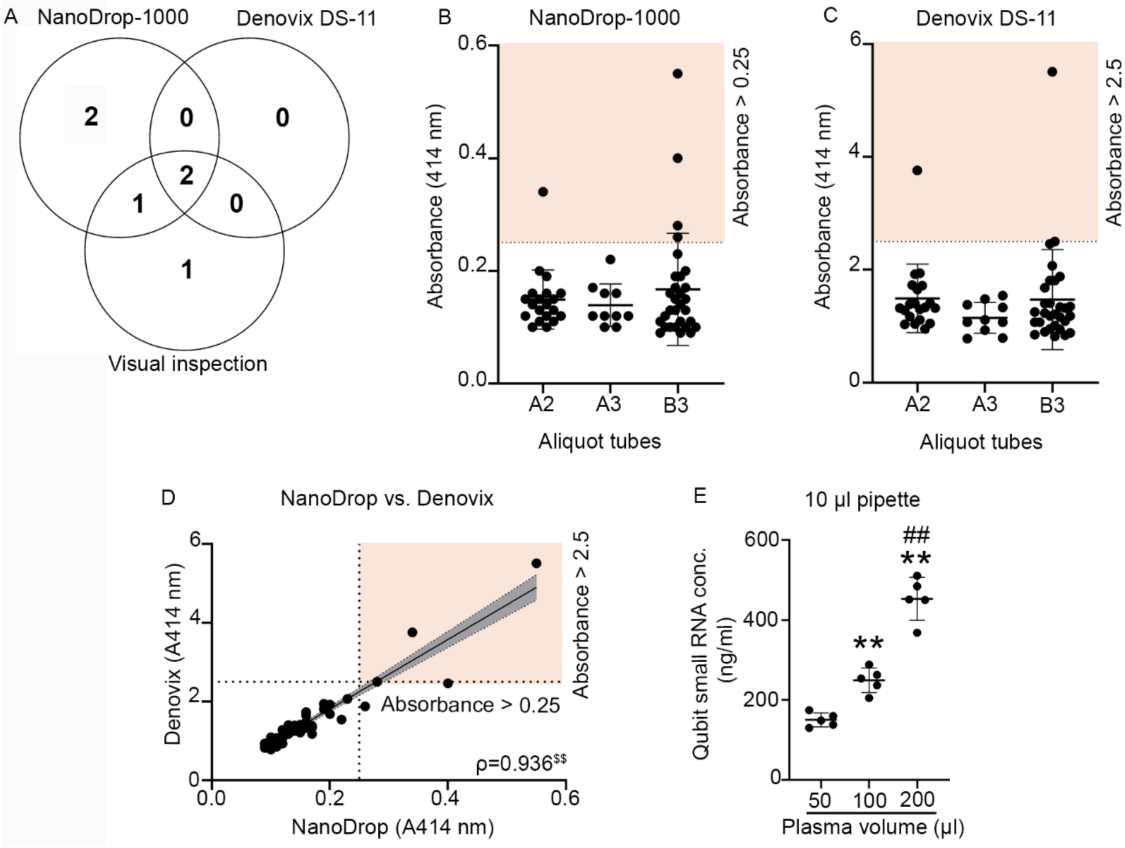

Supplementary figure 4. Plasma hemolysis analyses and small RNA concentration measurements in the validation phase. (A) 6/60 of the plasma aliquots were detected as hemolysed with at least one of the three methods and 2/60 with all the three methods. **(B-C)** The NanoDrop and Denovix revealed no difference in the mean hemolysis coefficients between the A2, A3 and B3 aliquots. **(A)** The NanoDrop and Denovix measurements were highly correlated (Spearman rho (ρ)=0.936, p<0.01). The orange shaded area in panels **(B-C)** indicate the samples with hemolysis absorbance values > 0.25 or > 2.5 in the NanoDrop and Denovix, respectively. Small RNA concentration measurements from the RNA extracted from 50-µl, 100-µl and 200-µl plasma volumes revealed increase in concentration with increase in starting plasma volumes. Statistical significances: $$p<0.01 in Spearman's correlation, **p<0.01 compared with the 50 µl plasma volume group, ##p<0.05 compared with the 100-µl plasma volume group. Abbreviations: A2, the 2nd 50-µl plasma aliquot from

the EDTA-A tube; A3, the 3rd 50-µl plasma aliquot from the EDTA-A tube; B3, the 3rd 50-µl plasma aliquot from the EDTA-B tube; conc., Concentration.

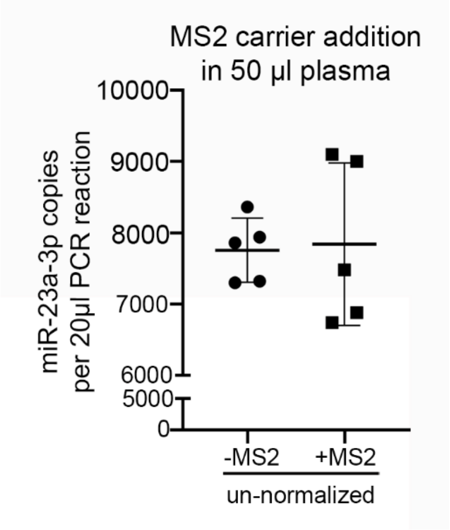

Supplementary figure 5. Effect of MS2 carrier addition on miR-23a-3p yield in the validation phase. Analysis of miR-23a-3p levels with miRCURY ddPCR from RNA extracted from 50-µl plasma volume revealed no significant improvement in the miRNA yield with MS2 carrier addition. Abbreviations: PCR, polymerase chain reaction.

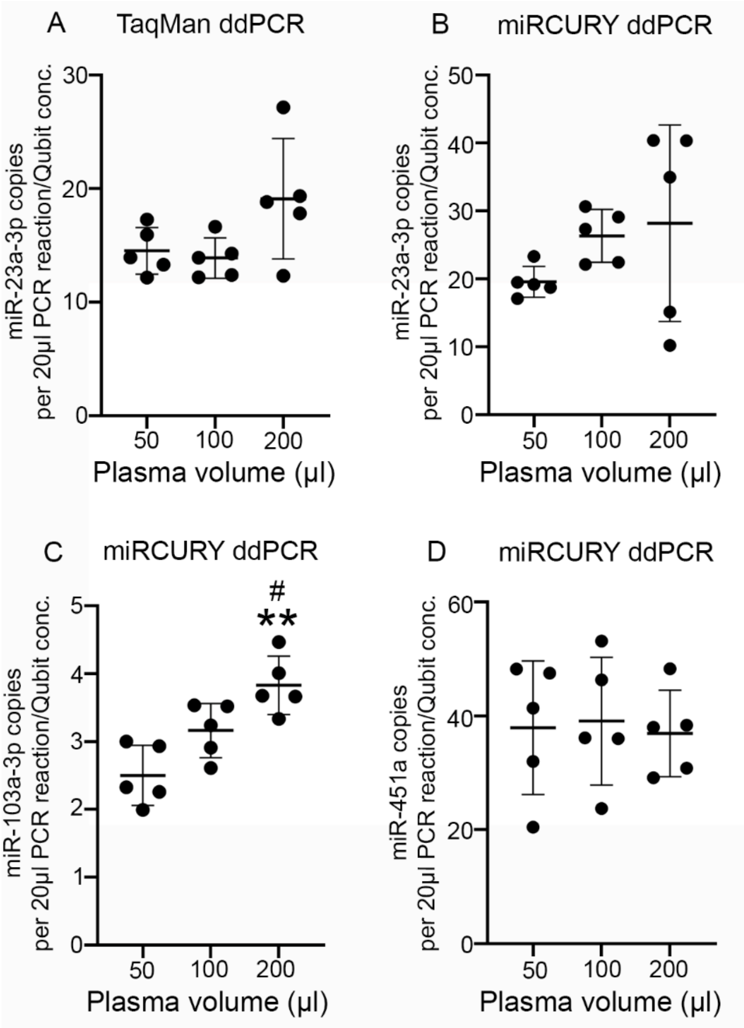

Supplementary figure 6. Posteriori normalization of miRNA levels from fixed RNA volume input to the small RNA concentration measured with Qubit. Posteriori normalization was performed by dividing the mean miRNA copy numbers obtained from TaqMan and miRCURY ddPCR of un-normalized samples by the small RNA concentration measured with Qubit. With this method, **(A-B)** the mean miR-23a-3p copies from TaqMan and miRCURY ddPCR normalized for all the three plasma volume groups. **(C)** Mean miR-103a-3p copies were still higher in the 200-µl plasma volume group in comparison to 50-µl and 100µl. **(D)** miR-451a levels also normalized for all the three plasma volume groups. Statistical significances: **p<0.01 compared to 50-µl group, #p<0.05 compared to 100-µl group. Abbreviations: conc., Concentration; PCR, polymerase chain reaction.
